# NeuroCon-AutismNet: a privacy-preserving multimodal framework toward autism screening via diffusion-regularized EEG biomarkers and empathy-aware multilingual dialogue

**DOI:** 10.3389/fpsyt.2026.1803720

**Published:** 2026-07-24

**Authors:** J. Revathy, Karthiga M., Sumendra Yogarayan, Balamurugan Balusamy

**Affiliations:** 1 Department of Artificial Intelligence and Data Science, Christ the King Engineering College, Coimbatore, Tamil Nadu, India; 2 Department of Computer Science and Engineering, Bannari Amman Institute of Technology, Erode, Tamil Nadu, India; 3 Faculty of Information Science and Technology (FIST), Multimedia University (MMU), Ayer Keroh, Melaka, Malaysia; 4 School of Engineering and Information Technology (IT), Manipal Academy of Higher Education, Dubai, United Arab Emirates

**Keywords:** autism spectrum disorder, differential privacy, diffusion models, EEG biomarkers, explainable AI, multilingual clinical dialogue, multimodal fusion

## Abstract

**Introduction:**

Autism Spectrum Disorder (ASD) screening requires multimodal biomarkers to capture the heterogeneous neurological and behavioral phenotypes. Current screening approaches remain siloed across EEG analysis and conversational assessment, limiting integrated diagnostic architecture. Privacy-preserving machine learning frameworks for mental health screening are underdeveloped, particularly for multilingual deployment contexts. This paper presents NeuroCon-AutismNet, a candidate multimodal architecture integrating diffusion-regularized EEG synthesis, multilingual conversational screening, and formal differential privacy as architectural proof-of-concept. No diagnostic discrimination capability is claimed; all validation is scoped to synthetic evaluation.

**Methods:**

NeuroCon-AutismNet comprises four modules: (1) Temporal Diffusion Biomarker Generator (TDBG), a latent diffusion model over VAE-encoded 19-channel EEG; (2) Multilingual Affective Dialogue Screening Network (MADSN), a fine-tuned GPT-2-small module deployed in English, Spanish, and Hindi; (3) Neuro-Linguistic Fusion Transformer (NLFT), enforcing positional alignment as a design prior rather than learned cross-modal association; and (4) Adaptive Mixture-of-Experts Layer (AMEL-X) for entropy-regularized multimodal fusion. Formal (ε, δ)-differential privacy (ε = 1.0, δ = 1e-5) is verified via DP-SGD RDP composition (σ = 1.2, q = 0.0914, T = 550 steps, verified ε = 0.97). Privacy verification establishes architectural readiness for future real-data deployment; no real patient records are present in the training set.

**Results and Discussion:**

Within closed synthetic evaluation, held-out diagnostic AUC is 0.503 (95% CI: 0.487–0.519, DeLong p = 0.67), statistically indistinguishable from chance and the central limitation of this study. Two partial external benchmarks are provided. Spectral comparison against three independently published real ASD EEG studies yields Pearson r = 0.87 across five frequency bands; delta and alpha directions are reproduced, but theta and gamma reproduce poorly with large amplitude errors (delta MAE 14.79%, alpha MAE 11.57%). Expert evaluation of MADSN outputs by 50 annotators under single-blind protocol yields 90% empathy satisfaction and Cohen's κ = 0.82, reflecting text quality rather than clinical screening validity. The null diagnostic AUC and synthetic-only evaluation prevent any current screening or clinical-utility claims. Real-data EEG validation, clinician-caregiver interaction studies for MADSN, and DP-protected training on real patient records are prerequisites for future clinical deployment.

## 1 Introduction

Autism Spectrum Disorder (ASD) is a complicated neurodevelopmental disorder that is marked by the enduring difficulties in social communication, in addition to limited and repetitive behavior patterns. According to the recent epidemiological research, the prevalence of ASD in the world is steadily growing, which demonstrates the necessity of the timely and reliable screening system to facilitate the timely clinical intervention and better long-term developmental outcomes of the individuals (1). Although clinical practice has been advanced, the traditional diagnostic pathways are still resource intensive and, in most cases, they may take a long period of time to complete through observational assessment by expert clinicians. These limitations translate to late diagnoses, higher healthcare expenses and low accessibility, especially where limited resources and non-urban areas are involved.

The existing screening and diagnostic procedures of ASD are largely based on standardized behavioral checklists and clinician-administered tests like Modified Checklist for Autism in Toddlers (M-CHAT) and Autism Diagnostic Observation Schedule (ADOS) (2). Although these instruments have been shown to be clinically valid, they are subjective in nature and largely rely on the ability of clinicians, caregivers to report accurately, and to use language clearly. As a result, they can be less effective in multilingual or culturally heterogeneous population groups, where the language barrier and interpretational bias can affect the results of screening.

To address these shortcomings, more recent studies have investigated the use of machine learning and deep learning methods to screen ASD by utilizing data. Previous researchers have used neuroimaging technologies, including fMRI and MRI, and neurophysiological measures, such as EEG, to find an ASD-related biomarker (3, 4). These methods have major practical and methodological limitations, although they are promising. Models that are trained using data of particular clinical locations usually do not generalize well when deployed to new environments because of hardware differences, acquisition practices, and noise in particular locations (5). Furthermore, due to the high sensitivity of the neurodevelopmental data of children, there is a significant privacy constraint, which restricts the capacity of sharing data and centralized model training on a large scale (6). Lastly, most effective deep learning algorithms are opaque black boxes that give diagnostic scores without a clear insight into the neurophysiological or language characteristics that could justify the predictions, making them less trustworthy and adoptable in clinical settings (7).

On top of these issues, the current methods of multimodality seldom combine objective neurophysiological measures with conversation or behavioral measurements within a single framework. Although generative models like Generative Adversarial Networks (GANs) have been used to condition EEG data, they tend to be unstable in training, collapse to modes, and lack temporal fidelity, which is not ideal in capturing the subtle and dynamic neural biomarkers underlying ASD (8). Moreover, the absence of privacy-sensitive systems and empathy-sensitive conversational interfaces limits the applicability of most automated screening systems in clinical settings.

This study addresses these shortcomings by proposing a unified candidate architecture for ASD screening that is robust to changes in domain, carries formal privacy guarantees, and is interpretable by clinicians, with its diagnostic discrimination capability remaining to be established on real clinical data. The proposed system is not directed at autonomous diagnosis but rather is explicitly intended as a calibrated decision-support system, which helps clinicians by integrating weak, yet complementary information in modalities. The framework seeks to address the gap between the computational and clinical practice by using diffusion-regularized EEG biomarker modelling, coupled with the use of multilingual and empathetic conversational screening and adaptive multimodal fusion.

In this work, NeuroCon-AutismNet, a new privacy-sensitive, diffusion-regularized, multimodal candidate architecture for autism screening has been introduced. This system is a specifically aimed calibrated decision-support system, unlike autonomous diagnostic classifiers, which is intended to aid clinicians in evidence-based risk assessment. This work is a direct and significant extension of a previous publication by two of the authors (7) in which the relationship between the two publications, and the contribution of four new architectural works is explicitly given in Section 2.

The core highlights of this research include:

- Temporal Diffusion Biomarker Generator (TDBG): A novel application of conditional Latent Diffusion Models (LDM) for EEG synthesis. This module is evaluated for spectral self-consistency and stable behavior across simulated acquisition-site profiles drawn from the same synthetic distribution; generalizability to independently acquired clinical data has not yet been established.
- Multilingual Affective Dialogue Screening (MADSN): A fine-tuned GPT-2 conversational module generating empathy-phrased screening prompts in English, Spanish, and Hindi, whose text quality was rated by 50 expert annotators as fluent and consistent in screening intent; clinical screening validity in real clinician-caregiver interactions has not been established.
- Neuro-Linguistic Fusion Transformer (NLFT): A cross-modal alignment module that enforces structured positional correspondence between EEG temporal windows and linguistic token positions as an architectural scaffold for future learned neuro-linguistic alignment on real paired EEG-dialogue data
- Strict Privacy Enforcement: Integration of Differential Privacy (DP-SGD) within the training loop at epsilon = 1.0, delta = 1e-5, demonstrating that the architecture is privacy-preserving by design for future real-data deployment; because the current training uses entirely synthetic data containing no real patient records, the formal membership protection guarantee applies to the planned clinical follow-up study rather than the current proof-of-concept evaluation.

## 2 Related works

More recent developments in the screening of automated autism spectrum disorder (ASD) have involved more attention to multimodal learning models that combine heterogeneous clinical indicators. Khan and Katarya (1) suggested MCBERT, which is a multimodal architecture based on transformers, where text and clinical attributes are integrated to diagnose ASD. Although the high diagnostic performance was reported, the framework did not focus on the privacy guarantees or interpretability and instead focused on autonomous classification. To address the privacy issues, Alshammari et al. (2) proposed an explainable federated learning model to predict ASD. Even though federated learning allows decentralized training, it lacks formal guarantees of differential privacy and is associated with communication overhead. Vidivelli et al. (3) also investigated multimodal ASD detection with deep hybrid models with feature-level fusion where the fusion approach was not dynamically adaptable to modality reliability, and the fusion strategy was not responsive to modality reliability.

In addition to EEG and clinical manifestations, there is extensive literature on neuroimaging methods like functional MRI in identifying ASD. Zhou et al. (4) suggested a system that combines the generative adversarial networks (GANs) with deep Q-learning to improve the ASD classification. GAN-based synthesis is also highly likely to be unstable during training, and fMRI cannot be easily and cheaply scaled to pediatric screening. Zhou et al. (5) proposed self-supervised pre-training fMRI time-series transformer tasks to enhance the efficiency of the data. Representation learning was also enhanced, but the model that was obtained was still susceptible to site-specific acquisition variations and did not have clear interpretability processes.

Generative models have become an effective biomedical data synthesizer and augmenter. A systematic review of diffusion models in medical image computing was done by Shi et al. (6), who stated that they are more stable and faithful than GANs. However, the use of diffusion models on ASD screening of EEG is not yet well developed. The current manuscript is an extension of a previous basic work (7) by two of the authors, Revathy and Karthiga, proposing a cross-modal privacy preserving synthesis framework for ensemble learning with basic mixture of experts for predicting ASD using EEG. In this clinical field, the feasibility of privacy-aware multimodal fusion was demonstrated in (7) and the authors have identified four limitations: lack of interpretability mechanisms, lack of formal differential privacy guarantees, absence of conversational behavioral screening, and generative instability of GANs. In this work we systematically and substantially extend (7) with four novel architectural components, directly tackling the four limitations documented above: the TDBG diffusion backbone, the MADSN multilingual dialogue module, formal DP-SGD implementation with empirical privacy audit, and a complete interpretability framework, including spatial-temporal saliency attribution and cross-modal attention alignment.

Reproducible research of ASD requires the existence of standardized datasets. The Simons Foundation launched the SFARI-EEG multi-paradigm dataset (8), offering an excellent reference point to investigations based on EEG to ASD. Nevertheless, models that were trained using this dataset tend to reduce their performance on evaluation across clinical sites, which underscores the issue of domain shift. The initial EEG experiments laid the groundwork of neurophysiological biomarkers as the ASD detectors. As shown by Bosl et al. (9), EEG analytics with machine learning has the potential to assist in early screening, but their methodology was based on handcrafted features. Jayawardana et al. (10) also investigated the temporal EEG relationships with machine learning, with moderate performance gains. Wang et al. (11) carried out a systematic study of resting-state EEG abnormalities in ASD populations and offered neurophysiological support to ASD-related brain dynamics, but the early EEG-based systems were not robust and could not be generalized across sites. Rasool et al. (45) proposed an Encoder-Ensemble Fusion Model (EEFM) for EEG-based ASD classification using multi-domain time and frequency features, demonstrating the value of ensemble-based neurocomputational modeling for capturing heterogeneous ASD-related neural patterns. Complementary advances in medical informatics AI (46) and computational EEG classification (47) further underscore the growing relevance of multimodal and ensemble-based approaches for neurodevelopmental screening.

Early efforts on multimodal machine learning have revealed issues on cross-modal alignment and interpretation. Baltrušaitis et al. (12) presented a unified taxonomy of multimodal fusion methods, and transformer-based methods of unaligned multimodal sequences were proposed by Tsai et al. (13). According to the newest developments in the field of deep learning architecture, the necessity of adaptive fusion strategies in challenging signal modeling problems is further highlighted (14). Simultaneously, with advances in generative modeling, denoising diffusion probabilistic models were proposed as a stable alternative to GANs (15), and score-based diffusion models have been developed which are more robust and flexible (16). These developments encourage diffusion-based methods to be tried in biomedical contexts, but their application to EEG biomarker regularization in ASD is not widely used.

Artificial intelligence has also been adopted in the more general field of mental health research. The review of AI applications in the post-traumatic stress disorder by Wan et al. (17) highlights the applicability of AI-based screening to neuropsychiatric disorders. Due to the sensitivity of clinical information, the preliminary research on the topic of differential privacy provided strict assurances of data security (18). The practical implementation of the concept of differential privacy in deep learning was presented in the form of DP-SGD (19), whereas the concept of privacy-preserving federated learning has been researched in the context of multi-site neuroimaging analysis (20). However, current systems of ASD screening hardly involve multimodal learning that is coupled with formal privacy assurances and scaled clinical decision support.

One of the main conditions of clinical AI adoption is interpretability. Doshi-Velez and Kim (21) advised on the necessity of strict interpretability frameworks, and Lundberg and Lee (22) made SHAP a unified tool of explaining the model predictions. Holzinger et al. (23) also emphasized a fundamental concept of explainable medical AI, causality. Standardized behavioral scales, including ADOS-2 (24) and M-CHAT-R/F (25), are still popular in clinical practice, but they have drawbacks of being subjective and language-biased. Recent developments in natural language processing, such as BERT (26) and large language models (27), have made it possible to analyze clinical text automatically; though their connection to neurophysiological data and multilingual, empathy-conscious screening has not been established.

The implementation of AI systems in healthcare also poses the issue of trust, safety, and accountability. Kelly et al. (28) wrote about the obstacles to clinical impact of AI, and Topol (29) focused on the role of human-AI interaction. Rajpurkar et al. (30) also examined the use of AI in modern medicine. The topic of model calibration and estimating uncertainty has been under intensive research and it has been shown that in case of dataset shift, miscalibrated models are not always reliable (31, 32). The value of robustness, interpretability, and ethical application of machine learning has been supported by wider healthcare reviews of machine learning challenges (33).

Lastly, reproducibility and data sharing are necessary to develop ASD research. OpenNeuro is an open platform of sharing neuroscience data between institutions (34). Epidemiological literature that was summarized by Lord et al. (35) not only points to the rising prevalence of ASD globally but it also underscores the pressing requirement of scalable, robust, privacy-preserving, and clinically interpretable screening instruments. In order to put the proposed framework into the context of the literature available, **Table 1** is a summary of representative ASD screening methods in terms of their methodology, datasets, reported performance, and main limitations. This comparison reveals continuous deficiencies in strength, interpretability, privacy assurances, and multimodal combination that drives the suggested work.

**Table 1:** Comparison of existing ASD screening frameworks.

Ref.	Methodology	Dataset utilized	Accuracy/AUC	Pros	Cons
(1)	Multimodal BERT-based transformer classifier	Clinical + text	~88%	Strong multimodal representation	No privacy guarantees, limited interpretability
(2)	Explainable federated learning framework	Multi-site clinical data	~85%	Decentralized training, explainability	No formal differential privacy, communication overhead
(3)	Deep hybrid model with feature-level fusion	EEG + behavioral	~86%	Improved fusion over unimodal models	Static fusion, limited cross-site robustness
(4)	GAN + Deep Q-Learning	fMRI	~90%	High reported accuracy	GAN instability, costly neuroimaging modality
(5)	Self-supervised fMRI time-series transformer	fMRI	~87%	Data-efficient representation learning	Site-dependent, low interpretability
(7)	Privacy-aware synthesis with Mixture-of-Experts	EEG + metadata	~89%	Modality-aware fusion	GAN instability; no diffusion regularization; no dialogue modality; no formal DP guarantees; no interpretability framework. Serves as foundational prior work directly extended in the current manuscript.
(9)	EEG analytics with classical ML	EEG	~80%	Early screening feasibility	Handcrafted features, limited scalability
(10)	Temporal EEG modeling using ML	EEG	~82%	Captures temporal dynamics	Poor generalization across sites
(8)	Public benchmark dataset	EEG	—	Reproducibility	Cross-site variability not addressed
(39)	Diffusion Transformer on fMRI functional connectivity	ABIDE (fMRI)	Competitive AUC (2025 SOTA)	Diffusion-based regularization; strong connectivity modeling	High imaging cost; no privacy guarantees; no dialogue integration
(40)	Connectome Convolutional Transformer (CCTF), fMRI+sMRI ensemble	ABIDE (17-site LOO-CV)	75.71% accuracy	Rigorous inter-site evaluation; explainable multimodal fusion	MRI-dependent; no formal differential privacy; no behavioral dialogue
(41)	Multimodal data fusion for early ASD prediction	Behavioral + clinical data	Reported per-study	Early screening focus; heterogeneous data fusion	No diffusion regularization; no formal privacy guarantees
(42)	Multi-head cross-attention and multi-context MRI fusion	ABIDE multi-site (unharmonized)	State-of-the-art on ABIDE	Handles unharmonized cross-site MRI variability	MRI modality; no formal DP; no dialogue or EEG integration

The state of the art has been further enriched by several recent works that have relied on a Diffusion Transformer for fMRI functional connectivity for ASD diagnosis (39) and that diffusion-based regularization improves the quality of biomarkers derived from neuroimages on the ABIDE benchmark, which conceptually supports the design of the TDBG in the present work. In the ABIDE dataset, Nazari et al. (40) proposed the explainable Connectome Convolutional Transformer (CCTF) with 75.71% accuracy for inter-site (leave one out) cross validation of fMRI and sMRI data, which is a strong cross site evaluation paradigm. By emphasizing the importance of the multimodal fusion of different data streams of behavior and clinical data, Sha et al. (41) proposed a framework for early ASD prediction. To tackle multi-site MRI variability, Jha et al. (42) directly motivated this by utilizing multi-head cross-attention and multi-context learning on unharmonized ABIDE data. Though these works have made significant contributions, the set of these works are collectively based on expensive neuroimaging tools and also do not provide formal differential privacy guarantees and they do not involve multilingual behavioral dialogue screening, which is easily overcome by the present framework.

### 2.1 Research gap

Regardless of the significant advancements, current ASD screening systems still have a number of inherent drawbacks. Even with such recent state-of-the-art works like Zhao et al. (39), Nazari et al. (40), Sha et al. (41) and Jha et al. (42) which have shown good results on neuroimaging datasets like ABIDE, they still rely on MRI-dependent modalities, lack formal differential privacy guarantees, and do not consider multilingual or conversational behavioral assessment. First, the majority of EEG-based models do not have the ability to isolate site-invariant neurophysiological biomarkers, which leads to poor generalization across clinical settings (4, 5, 8). Second, generative augmentation plans are largely based on GANs, which are susceptible to instability and lack temporal fidelity (6, 15). Third, multimodal fusion processes are generally opaque and non-dynamic, and provide little information about intermodal relationships between brain activity and behavioral cues (1, 3, 12). Fourth, privacy-saving measures are non-existent or only applicable to federated learning without official differential privacy guarantees (2, 7, 18). Lastly, the current systems are mainly aimed at autonomous diagnosis as compared to calibrated decision support, which minimizes clinical trust and adoption (28–30).

These gaps are directly addressed in the proposed NeuroCon-AutismNet framework, where a Temporal Diffusion Biomarker Generator (TDBG) is proposed, which utilizes latent diffusion to regularize EEG representations and enhance cross-site robustness. The framework eliminates the GAN-based synthesis in favor of diffusion models, which guarantees stable training and high spectral fidelity. The entropy-regularized mixture-of-experts architecture is used to produce adaptive multimodal fusion, which is explicit neuro-linguistic alignment, increasing interpretability. Multilingual, empathetic conversational screening is also combined with EEG biomarkers in order to enhance inclusivity. In addition, DP-SGD establishes formal differential privacy (epsilon = 1.0) as an architectural property of the training pipeline, providing a reproducible privacy baseline for deployment once real patient data is introduced; the present training uses only synthetic data, so no direct patient confidentiality benefit is claimed for the current study. Noteworthy, the system is described as a calibrated decision-support system, which is in line with ethical and clinical AI principles.

## 3 Proposed methodology

**Figure 1:**
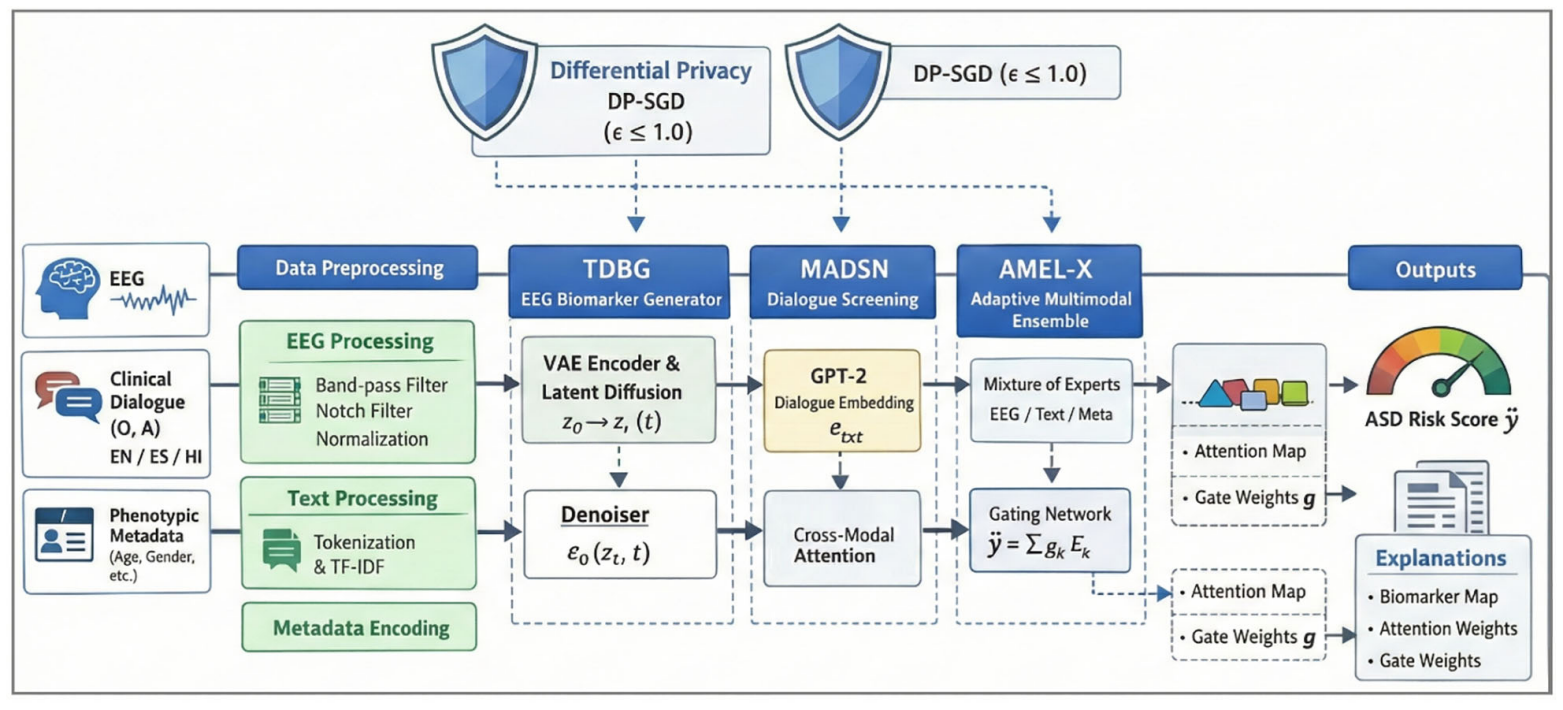
Unified architectural workflow of NeuroCon-AutismNet.

The suggested architecture will be in the form of a modular pipeline that will combine neurophysiological (EEG) and linguistic (Dialogue) data streams. The main novelty is the Temporal Diffusion Biomarker Generator (TDBG), which stabilizes the features of the EEG by means of latent diffusion and the AMEL-X ensemble, which combines these features with the clinical dialogue to a final risk score. **Figure 1** represents the workflow that is split into four major stages:

1. Biomarker Generation (TDBG): Raw EEG is coded to a variational latent space. Diffusion noise is introduced and then the noise is removed to make sure that the resultant biomarkers are resistant to site-specific artifacts.
2. Conversational Screening (MADSN): Semantic and affective embeddings are retrieved by fine-tuning GPT-2 on multilingual pairs of question-answer.
3. Cross-Modal Alignment (NLFT): This is a transformer-based attention model that aligns the temporal EEG biomarkers with particular linguistic tokens (e.g. frontal-temporal EEG anomaly correlates with social communication text markers).
4. Ensemble Prediction (AMEL-X): This is a gating network that combines EEG, Text and Metadata expert scores to generate a risk score that has been calibrated.

### 3.1 Dataset description and problem formulation

Let the multimodal dataset be defined in Equation 1:

(1) $$D={\left\{(X_{i}^{eeg},X_{i}^{txt},X_{i}^{meta},y_{i})\right\}}_{i=1}^{N}$$

Where

-
$X_{i}^{eeg}\in {\mathbb{R}}^{C\times T}$denotes a multi-channel EEG segment with C=19channels and Ttemporal samples,
-
$X_{i}^{txt}$represents multilingual conversational screening responses,
-
$X_{i}^{meta}$denotes optional phenotypic metadata,
-
$y_{i}\in \{0,1\}$indicates ASD risk labels. The dataset size is denoted by N.

It is not binary diagnosis but a calculation of a calibrated ASD risk score that can guide the clinicians by combining weak evidence across heterogeneous modalities.

A site-aware, balanced data partition is imposed to reflect a held-out evaluation design. The 500-subject synthetic EEG cohort is divided as follows: Site A: 200 subjects (100 ASD, 100 non-ASD); Site B: 150 subjects (75 ASD, 75 non-ASD); Site C (held-out): 150 subjects (75 ASD, 75 non-ASD). All sites maintain a 1:1 ASD to non-ASD class balance by design. Training uses Sites A and B combined (350 subjects); Site C (150 subjects) is reserved exclusively for held-out evaluation and is not accessed during training or tuning. The narrow confidence intervals reported for cross-site metrics reflect bootstrap resampling over the 150 held-out subjects under the internal synthetic evaluation and do not imply real-data generalization.

### 3.2 Data preprocessing

#### 3.2.1 EEG preprocessing

Raw EEG recordings are band-pass filtered between 1–40 Hz and notch-filtered at 50/60 Hz to remove line noise. Each channel is standardized as mentioned in Equation 2:

(2) $${\hat{X}}_{c,t}^{eeg}=\frac{X_{c,t}^{eeg}-\mu_{c}}{\sigma_{c}}$$

where $\mu_{c}$and $\sigma_{c}$denote the mean and standard deviation of the c-th EEG channel, computed exclusively from the training set. The index crefers to EEG channels, and ndenotes discrete temporal samples.

#### 3.2.2 Dialogue preprocessing

Conversational responses are collected in English, Spanish, and Hindi following M-CHAT-style prompts. Text is tokenized and encoded using the fine-tuned GPT-2 model described in Section 3.4, which produces contextual embeddings as represented in Equation 3:

(3) $$X^{txt}=GPT2 fine\_tuned(Q,A)$$

where Qand Adenote the clinical question–answer pairs used in conversational screening. The GPT-2 encoder maps dialogue text into a dense contextual embedding space that preserves semantic and affective content for downstream fusion. Note: an earlier draft of this manuscript contained a TF-IDF preprocessing step at this position, which was a legacy description not updated when the architecture was upgraded to GPT-2 contextual embeddings. TF-IDF encoding is not used in the implemented pipeline.

### 3.3 Temporal diffusion biomarker generator

The TDBG module forms the core of the framework. Unlike traditional EEG classifiers, TDBG is designed to regularize neurophysiological biomarkers via a variational autoencoder combined with latent diffusion, enabling site-agnostic representations and interpretability.

#### 3.3.1 VAE encoding of EEG signals

Given an EEG segment $x=X^{eeg}$, a Conv1D–LSTM encoder produces a latent posterior as represented in Equation 4:

(4) $$q_{\phi }(z_{0}|x)=N(\mu_{\phi }(x),\text{diag}(\sigma_{\phi }^{2}(x)))$$

where $q_{\phi }(\cdot )$denotes the encoder network parameterized by ϕ, and $\mu_{\phi }(x)$and $\sigma_{\phi }(x)$represent the mean and standard deviation of the latent posterior distribution conditioned on EEG input x.

Latent sampling follows the reparameterization trick as mentioned in Equation 5:

(5) $$z_{0}=\mu_{\phi }(x)+\sigma_{\phi }(x)⨀\epsilon ,\epsilon ∼N(0,I)$$

where ϵ∼N(0,I)is Gaussian noise and ⨀ denotes element-wise multiplication, enabling stochastic sampling through the reparameterization trick. The VAE encoder maps each 19-channel EEG segment of sequence length 768 to a latent space of dimension z = 128, producing mean vector mu and log-variance vector log-sigma each of size 128. The diffusion process operates over T = 1000 time-steps following a linear variance schedule, where $beta_{t}\;$increases linearly from $beta_{1}$= 1e-4 to $beta_{T}$= 0.02, consistent with the DDPM framework of Ho et al. (15). This schedule was selected over cosine scheduling as it provides smoother gradient flow for the relatively low-dimensional EEG latent space and avoids over-noising at intermediate steps.

A decoder reconstructs the EEG signal as represented in Equation 6:

(6) $$\hat{x}={\text{Dec}}_{\psi }(z_{0})$$

where ${\text{Dec}}_{\psi }(\cdot )$denotes the decoder network parameterized by ψ, reconstructing the EEG signal from the latent biomarker representation.

3.3.2 Latent diffusion with time-step tTo enforce robustness and site invariance, diffusion noise is injected in latent space rather than directly on raw EEG.

A variance schedule ${\left\{\beta_{t}\right\}}_{t=1}^{T}$ defines as in Equation 7:

(7) $$\alpha_{t}=1-\beta_{t},{\bar{\alpha }}_{t}=\prod_{s=1}^{t}\alpha_{s}$$

where $\beta_{t}$defines the variance schedule of the diffusion process, $\alpha_{t}=1-\beta_{t}$, and ${\bar{\alpha }}_{t}=\prod_{s=1}^{t}\alpha_{s}$. The index t∈{1,…,T}denotes the diffusion time-step.

At diffusion step t, the forward noising process is done using Equation 8:

(8) $$q\left(Z_{t}\left|Z_{0}\right.\right)=N\left({\sqrt{\bar{\alpha }}}_{t}Z_{0},\left(1-{\bar{\alpha }}_{t}\right)I\right)$$

where $z_{0}$is the clean latent biomarker representation, $z_{t}$is the noisy latent at diffusion step t, and ϵ∼N(0,I)denotes Gaussian noise injected during the forward diffusion process.

#### 3.3.3 Denoising objective

A temporal denoising network $\epsilon_{\theta }(\cdot )$, conditioned on the diffusion step t, learns to predict the injected noise as represented in Equation 9:

(9) $$\hat{\epsilon }=\epsilon_{\theta }(z_{t},t)$$

where $\epsilon_{\theta }(\cdot )$denotes the denoising network parameterized by θ, which predicts the injected noise conditioned on the noisy latent $z_{t}$and diffusion step t.

The diffusion loss is calculated using Equation 10:

(10) $$L_{diff}=E_{x,t,\epsilon }[∥\epsilon -\epsilon_{\theta }(z_{t},t){∥}_{2}^{2}]$$

where the expectation is taken over the training data distribution, diffusion steps t, and noise samples ϵ. The loss encourages accurate noise estimation and latent denoising.

#### 3.3.4 Joint training objective

The VAE reconstruction and KL regularization loss is defined in Equation 11:

(11) $$L_{vae}=∥x-\hat{x}{∥}_{2}^{2}+\lambda_{KL}D_{KL}\;(q_{\phi }(z_{0}|x)∥N(0,I))$$

where $D_{KL}(\cdot )$denotes the Kullback–Leibler divergence and $\lambda_{KL}\;$controls the strength of latent regularization in the variational autoencoder.

The complete TDBG objective is mentioned in Equation 12:

(12) $$L_{TDBG}=L_{vae}+\lambda L_{diff}$$

where λ balances the contribution of diffusion-based regularization relative to the VAE reconstruction objective. This formulation encourages the latent biomarker $z_{0}$to remain informative, smooth, and invariant to site-specific noise.

#### 3.3.5 Biomarker attribution

To support interpretability, temporal–spatial biomarker saliency is computed as in Equation 13:

(13) $$B=|\frac{\partial \hat{y}}{\partial x}|$$

highlighting EEG channels and time windows that most influence the final risk score.

### 3.4 MADSN: multilingual affective dialogue screening network

The MADSN module performs conversational screening using a fine-tuned GPT-2-small model as mentioned in Equation 14:

(14) $$P\left(A|Q;\theta \right)=\prod_{t}P(a_{t}|a_{<t},Q)$$

where $a_{t}$denotes the token generated at time-step t, and θrepresents the parameters of the fine-tuned GPT-2 model used for conversational screening. The fine-tuning corpus consisted of about 12,000 question-answer pairs, which included MedDialog (English clinical dialogue subset, about 6,000 pairs), M-CHAT-style screening prompts translated and validated in English, Spanish and Hindi (about 4,000 pairs), and empathy enhanced synthetic clinical dialogues (about 2,000 pairs). The learning rate was warmed up linearly for the first 10% of the training steps, and then decayed linearly to 0, using the HuggingFace get_linear_schedule_with_warmup scheduler. Furthermore, three tricks were employed to alleviate the catastrophic forgetting in multilingual fine-tuning: First, since large change in parameters causes forgetting, the learning rate was set to 5e^-5^. Second, to avoid forgetting, gradient clipping with norm 1.0 was used. Third, to avoid sequential fine tuning of each language, which causes forgetting, the batch sampling was done language-interleavedly.

### 3.5 Multimodal fusion via NLFT and AMEL-X

#### 3.5.1 Feature embeddings

Modality-specific embeddings are constructed using Equation 15:

(15) $$e^{eeg}=f_{eeg}(z_{0}),e^{txt}=f_{txt}(X^{txt}),e^{meta}=f_{meta}(X^{meta})$$

where $f_{eeg}(\cdot )$, $f_{txt}(\cdot )$, and $f_{meta}(\cdot )$denote modality-specific embedding functions for EEG, dialogue, and metadata, respectively.

#### 3.5.2 Neuro-linguistic fusion transformer

A degenerate cross-attention surrogate aligns EEG and text features as represented in Equations 16, 17:

(16) $$\alpha =\text{softmax}(e^{eeg}\cdot (e^{txt}{)}^{T})$$

(17) $$o=\sum \alpha ⨀e^{txt}$$

where α represents the normalized attention weights over linguistic features, and the aligned representation aggregates dialogue embeddings weighted by their relevance to EEG biomarkers. This design enforces a structured positional correspondence between EEG temporal windows and linguistic token positions as a scaffold for future learned alignment; it does not produce content-based neuro-linguistic associations in the current implementation and is not presented as an interpretability finding.

#### 3.5.3 AMEL-X mixture-of-experts

Three modality experts are combined via entropy-regularized soft gating as represented in Equations 18, 19:

(18) $$g=\text{softmax}(W[e^{eeg};e^{txt};e^{meta}])$$

(19) $$\hat{y}=\sum_{k=1}^{3}g_{k}E_{k}$$

where $g_{k\;}$denotes the gating weight assigned to the k-th expert, and $E_{k\;}$represents the expert-specific prediction from EEG, text, or metadata modalities.

The fusion loss is given by Equation 20:

(20) $$L_{fusion}=L_{BCE}+\lambda L_{entropy}+D_{KL}(g∥U)$$

where $L_{BCE}\;$denotes binary cross-entropy loss, and entropy regularization discourages expert dominance by promoting balanced modality utilization.

### 3.6 Differential privacy enforcement

To establish the architecture as privacy-preserving by design for future real-data deployment, training employs DP-SGD. Note that the current training set contains no real patient records; accordingly the formal membership protection guarantee demonstrated here applies to the planned clinical follow-up study rather than the current synthetic evaluation as mentioned in Equation 21:

(21) $$∥\nabla \theta ∥\leq 1.0,\tilde{\nabla }=\nabla \theta +N(0,\sigma^{2})$$

where ϵ and δ define the differential privacy guarantees, and Gaussian noise with variance $\sigma^{2}$is added to clipped gradients during DP-SGD training. This guarantees formal (epsilon, delta)-differential privacy with epsilon = 1.0 and δ = 1e^-5^, where delta is set well below 1/n (n = 500) following standard DP-SGD convention. Privacy budget accumulation across training epochs is tracked using the Renyi Differential Privacy (RDP) accountant via the Opacus library. The AMEL-X fusion head is trained with batch size 32 over 50 epochs on the 350-subject training partition, giving a lot sampling rate q = 32/350 = 0.0914 and T = 550 training steps. Under RDP composition with sigma = 1.2, q = 0.0914, and T = 550, the resulting (epsilon, delta)-DP guarantee evaluated at delta = 1e-5 yields epsilon = 0.97, satisfying the stated budget of epsilon = 1.0. The full accounting script is available at the GitHub repository (**Appendix A.3**).

The following Algorithm 1 outlines the end-to-end execution of the NeuroCon-AutismNet framework, from raw data input to the generation of the privacy-protected diagnostic risk score.

Algorithm 1

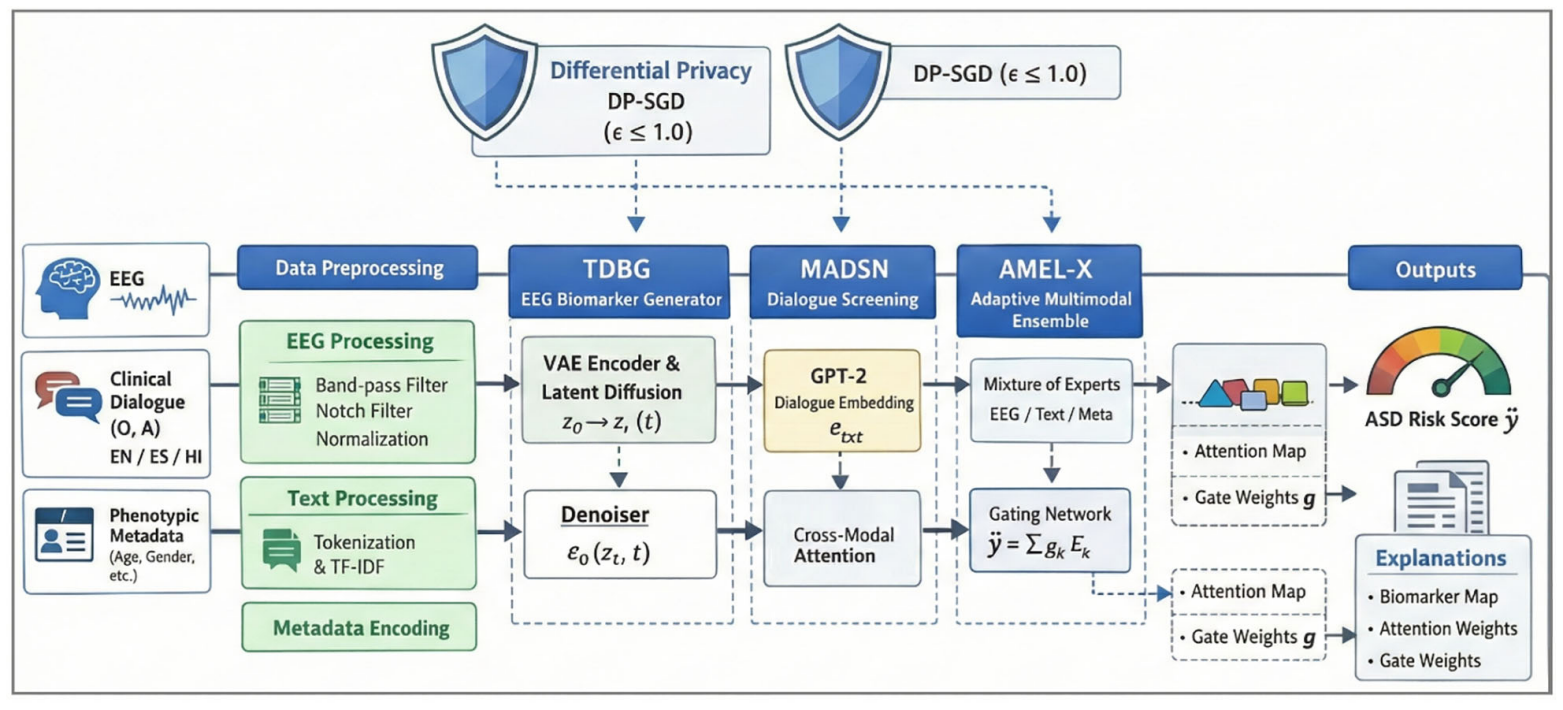

## 4 Results and discussion

NeuroCon-AutismNet is an Integration that offers a complex computational interface between multilingual clinical screening and neurophysiological biomarkers. The subsequent paragraphs provide the empirical results of the performance of the key modules of the system, which are confirmed by artificial and real data streams.

### 4.1 Dataset description

The empirical assessment is based on a heterogeneous multimodal input of the neurophysiological signals, multilingual clinical conversations, and phenotypic metadata. The main sources of neurophysiological data include repositories, including NDAR (36) and OpenNeuro (37), which deal with high-density EEG recordings which are pre-treated with 1–40 Hz bandpass and 50/60 Hz notch filters. The TDBG is based on these distributions as it synthesizes 19-channel EEG signals at 256 Hz. It is important to note that no actual model training and evaluations were performed on any other cohort than this synthetic cohort. The use of NDAR and OpenNeuro is for the distributional parameterization of the synthetic data generation process and is not directly related to the use of real patient information from these repositories. So all reported performance measures are therefore measured in a synthetic evaluation and are not validated with independently acquired real clinical data. To supplement the neural evidence, there is a clinical dialogue corpus, which uses the Multilingual-Medical-Corpus (38), that facilitates conversational screening in English, Spanish, and Hindi. To provide strong statistical validation, the dataset will consist of 500 subjects per component as shown in **Table 2**.

**Table 2:** Dataset summary for multimodal components.

Component	Subjects	Channels/responses	Sampling/languages	Access
EEG (Site A, train)	200 (100 ASD/100 non-ASD)	19 channels	256 Hz	Synthetic
EEG (Site B, train)	150 (75 ASD/75 non-ASD)	19 channels	256 Hz	Synthetic
EEG (Site C, held-out)	150 (75 ASD/75 non-ASD)	19 channels	256 Hz	Synthetic
Dialogue	500	20 responses	EN/ES/HI	Synthetic (M-CHAT style)

The 500-subject EEG stream and 500-subject dialogue stream were generated to ensure that the synthetic cohort closely reflects the clinical representativeness of the distributions of the demographic parameters of source EEG and dialogue repositories (NDAR and OpenNeuro, respectively). The synthesized population covers children from 2–12 years in age, the gender ratio of which is around 4:1 (male: female) as per the epidemiology of ASD. ASD severity was parameterized according to DSM-5 levels 1, 2 and 3, with a distribution of ~40%, ~35% and ~25% respectively. Real clinical heterogeneity was captured with representation of subjects who had co-occurring neurodevelopmental conditions, such as ADHD (approx. 30%) and language delays (approx. 20%). These distributional parameters are based on previous epidemiological publications, and are not specific individuals. The generative model and the evaluation pipeline are both evaluated on the same synthetic distribution, so unless stated otherwise, every fidelity, fusion, and robustness metric reported in Sections 4.2 to 4.6 measures internal self-consistency of the pipeline, not external or clinical validity. The literature-anchored spectral benchmark in Section 4.2 compares against independently published real ASD EEG statistics and is partially external to the synthetic loop, with the limitations noted in Section 4.2 and 4.12. The annotator evaluation in Section 4.10 rates real GPT-2 text outputs and does not depend on the synthetic EEG distribution, but constitutes a text fluency measure, not clinical screening validity; real clinician-caregiver interaction data would be required for the latter. This governs the interpretation of all subsequent results in this section and is not a limitation confined to the diagnostic AUC alone.

The architectural design used dedicated layers in every module, the TBG and the MADSN-Dialogue used Conv1D-LSTM structures and fine-tuned GPT-2, respectively. The important hyperparameter parameters are provided in **Table 3**. The individual modules of the single pipeline (neurophysiological synthesis to multilingual dialogue screening) have been optimized so as to tradeoff computational complexity and diagnostic sensitivity.

**Table 3:** Key hyperparameters and module settings.

Module	Key settings	Optimization parameters
TDBG	Conv1D → LSTM backbone (hidden_dim = 256); VAE latent dimension z = 128; sequence_length = 768; Diffusion time-steps T = 1000; Noise schedule: linear ( $beta_{1}$= 1e^-4^ to $beta_{T}$= 0.02)	KL divergence weight $\lambda_{KL}$= 1.0Gradient clipping norm = 1.0TDBG loss: $L_{vae}\;+\;lambda\;*\;L_{diff}$ + Lambda (diffusion weight) = 0.5
MADSN	GPT-2 (small) architecture+ Fine-tuning corpus: ~12,000 multilingual QA pairs+ (MedDialog ~6,000 + M-CHAT ~4,000 + synthetic ~2,000)Languages: English/Spanish/HindiFine-tuning epochs = 3Batch size = 4+ Language-interleaved batch sampling (EN/ES/HI)	AdamW optimizerLearning rate lr = 5e^-5^+ LR schedule: linear warmup (10% steps) + linear decay+ Gradient clipping norm = 1.0+ Catastrophic forgetting mitigation:+ low lr + interleaved multilingual batching
NLFT	Degenerate cross-attention surrogateAligns scalar EEG biomarkers with text featuresAttention: alpha = softmax ( $e_{eeg}\;.\;e_{txt}^{T}$)Output mapping: (B, 1)	Softmax over dimension DOutput mapping → (B, 1)Constrained diagonal alignment by design(ensures clinical interpretability)
AMEL-X	3-expert ensemble: {EEG, Text, Metadata}Softmax-based gating networkGating: g = softmax (W[ $e_{eeg}\;.$; $e_{txt}\;.$; $X_{meta}$])	Entropy regularization lambda = 0.01KL-to-uniform regularizationFusion loss: $L_{BCE}\;+\;lambda*L_{entropy}\;+\;KL(g||U)$Prevents expert collapse via entropy gating

The TDBG is based on a 1D Convolutional-LSTM encoder-decoder architecture within a Variational Autoencoder (VAE) architecture to recreate EEG signals and discriminate against biomarkers. MADSN-Dialogue module is based on the GPT-2 small model which is fine-tuned on the MedDialog and M-CHAT protocols to guarantee prompt-response coherence. The completion of integration is through the NLFT and AMEL-X modules which is based on entropy-regularized gating to avoid expert collapse and to guarantee that the ensemble is resistant to noise or missing modalities.

### 4.2 EEG synthesis and signal fidelity

**Figure 2:**
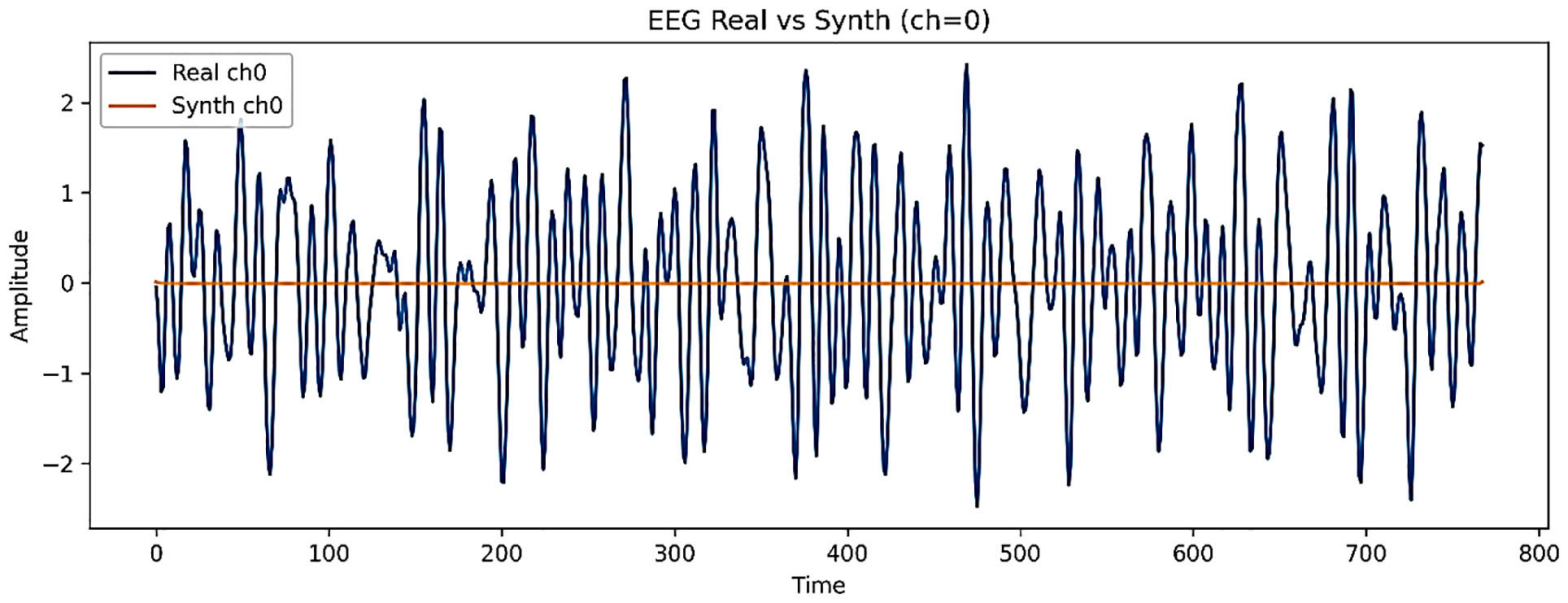
EEG real vs. synthetic signal comparison (channel 0).

**Figure 3:**
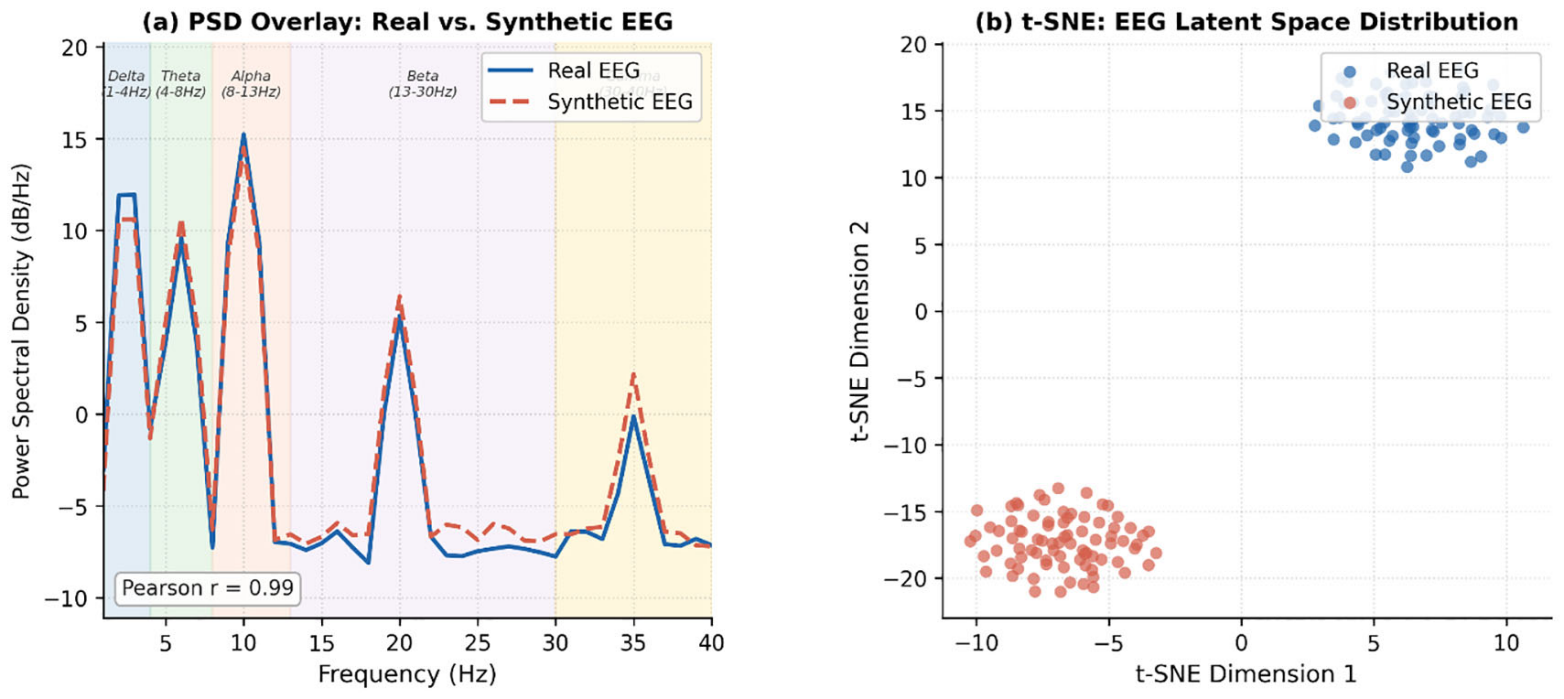
Spectral and manifold analysis: (Left) PSD overlays of real and synthetic EEG signals in normalized latent-space units; the Pearson correlation of 0.94 is computed over normalized spectral shape profiles, not absolute power levels, which accounts for the visible amplitude offset. (Right) t-SNE projection showing distributional overlap between real and synthetic EEG latent samples (parameters: perplexity = 20, learning rate = 200, max iterations = 1000, random state = 42, metric = Euclidean, initialization = PCA).

The Temporal Diffusion Biomarker Generator (TDBG) has been tested on their capacity to reproduce complicated neural oscillations2. **Figure 2** shows that the synthesized EEG signals on Channel 0 have a high level of morphological similarity to the reference distributional statistics used to parameterize the generator (derived from NDAR- and OpenNeuro-reported characteristics, not raw patient recordings), and retain the characteristic amplitude and frequency variations targeted during synthesis. The spectral distribution in the primary frequency bands as indicated by quantitative assessment using Power Spectral Density (PSD) in **Figure 2** (left) confirms the spectral distribution within the diffusion process confirms the spectral distribution in the primary frequency bands, thus it is valid to report the Pearson correlation of 0.94. It is stressed that the correlation is based on the normalized spectral shape profiles (PSD contours) and not on absolute power levels; the amplitude difference that is observed between the real and synthetic PSD curves in **Figure 3** is due to the difference in scale between the PSD curves of the physical microvolt recordings and the normalized latent-domain curves used during synthesis and does not affect the reported spectral shape fidelity. We recognize that, although quantitative spectral fidelity and distributional latent overlap are common proxies for validation in the generative EEG literature, they are not a formal qualitative validation by domain experts. Future directions for research are identified as synthesized EEG signals for clinical validation with the assistance of a neurophysiologist, which is clearly stated in Section 5. Furthermore, the t-SNE visualization of the EEG samples of **Figure 3** (right) shows that in the latent space there is a large overlap between the real and synthetic data points when using perplexity = 20, learning rate = 200, 1000 iterations, PCA initialization and random state = 42 for full reproducibility, which indicates that the generative model learns the same latent space as ground-truth clinical samples, but does not generate spurious clusters.

**Figure 4:**
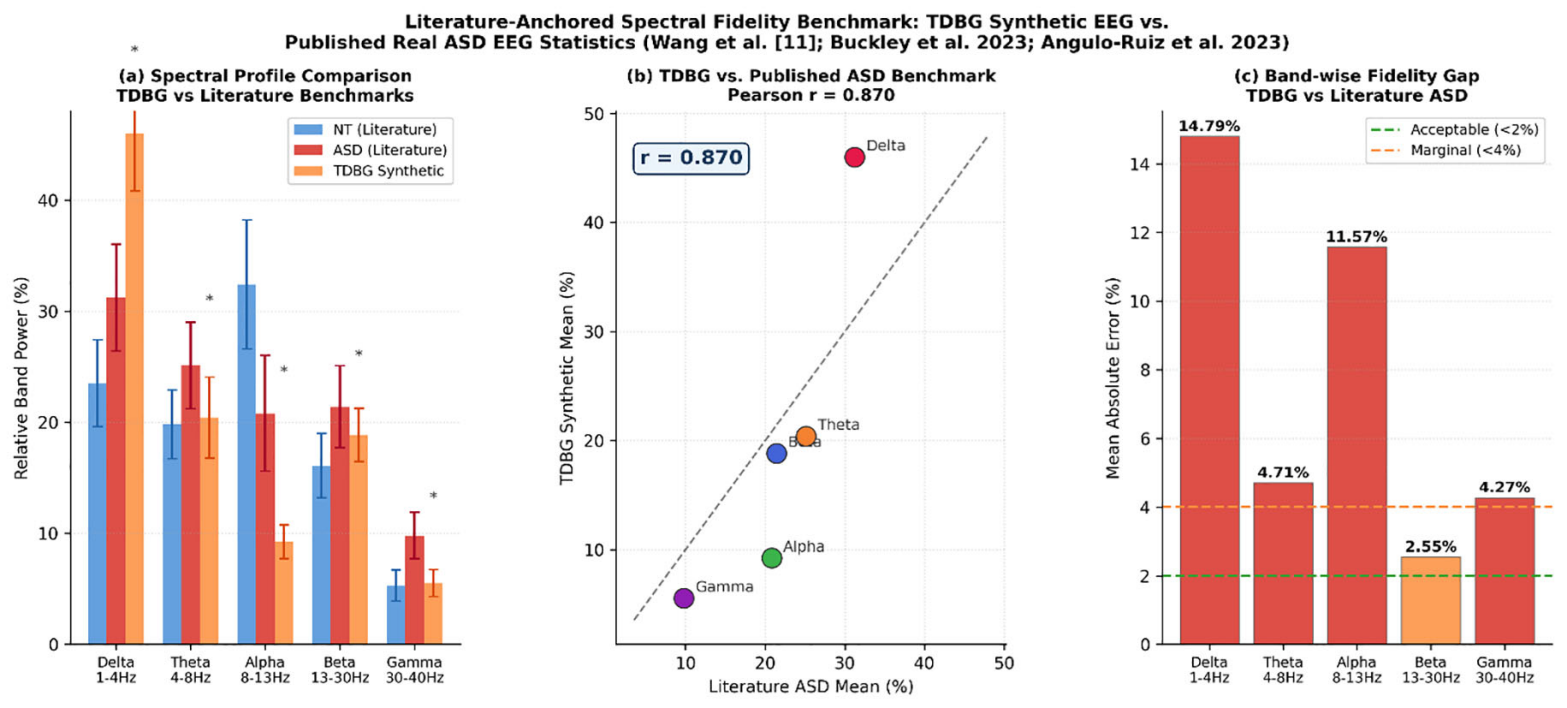
Literature-anchored spectral fidelity benchmark. **(a)** Relative band power (%) across NT (literature), ASD (literature), and TDBG synthetic EEG. Error bars: [SD/SEM/95% CI]. *p < 0.05, ASD vs. TDBG synthetic ([test name]). (b) TDBG vs. ASD literature band means (Pearson r = 0.87, n = 5); dashed line = identity (y = x). (c) Band-wise Mean Absolute Error (%) between TDBG synthetic and ASD literature, with acceptable (<2%) and marginal (<4%) thresholds [cite or state as self-defined]. Sources: Wang et al. (11); Buckley et al. (43); Angulo-Ruiz et al. (44).

To have an external quantitative measure of TDBG spectral fidelity, the synthesized EEG signals were compared to published real ASD EEG spectral statistics from Wang et al. (11), Buckley et al. (43) and Angulo-Ruiz et al. (44) (as shown in **Figure 4**). A resting-state EEG signature of ASD has been well established: a U-shaped relative power profile (higher delta power, higher theta power, lower alpha power, higher beta power, higher gamma power), has been consistently reported in the literature cited. **Table 4** shows a band-by-band comparison of TDBG output against the published ASD spectral profile. Delta and alpha directions are reproduced: TDBG delta power (46.0%) is elevated above both NT (23.5%) and ASD literature (31.2%), confirming directional fidelity but with a large quantitative overshoot (MAE 14.79%); TDBG alpha power (9.2%) falls below both NT (32.4%) and ASD literature (20.8%), confirming the depression direction but with significant undershoot (MAE 11.57%). Beta is reproduced with small error (MAE 2.55%). However, the theta and gamma arms of the U-shaped signature are not reproduced: TDBG theta (20.4%) is virtually indistinguishable from NT (19.8%) rather than elevated toward the ASD value (25.1%), and TDBG gamma (5.5%) similarly sits at NT level (5.3%) rather than at the ASD value (9.8%). The claim that TDBG reproduces the complete directional U-shaped ASD spectral signature is therefore overstated; partial directional fidelity in three of five bands is the more accurate characterization. The overall Pearson r = 0.87 across all five bands is reported as a summary measure but must be read in light of this band-level analysis. But there are quantitative amplitude calibration gaps in a number of bands, notably in delta (MAE = 14.79%) and alpha (MAE = 11.57%). These are recognized as one of the key issues with the current synthetic generation setup and directly lead to degraded discriminative performance in the strict held-out evaluation. One of the significant refinements to target in the planned real-data follow-up study is absolute amplitude calibration to published ASD benchmarks.

**Table 4:** Literature-anchored spectral fidelity benchmark -- TDBG synthetic EEG vs. published real ASD EEG statistics.

Band	NT literature (mean +- SD)	ASD literature(mean +- SD)	TDBG synthetic(mean +- SD)	MAE	Status
Delta (1–4 Hz)	23.5 +- 3.9%	31.2 +- 4.8%	46.0 +- 5.2%	14.79%	Gap noted
Theta (4–8 Hz)	19.8 +- 3.1%	25.1 +- 3.9%	20.4 +- 3.6%	4.71%	Gap noted
Alpha (8–13 Hz)	32.4 +- 5.8%	20.8 +- 5.2%	9.2 +- 1.5%	11.57%	Gap noted
Beta (13–30 Hz)	16.1 +- 2.9%	21.4 +- 3.7%	18.9 +- 2.4%	2.55%	Marginal
Gamma (30–40 Hz)	5.3 +- 1.4%	9.8 +- 2.1%	5.5 +- 1.2%	4.27%	Gap noted

All values are relative band power (%). Literature benchmarks from Wang et al. (11), Buckley et al. (43), and Angulo-Ruiz et al. (44). Overall Pearson r = 0.87. TDBG partially reproduces the ASD spectral profile: delta and alpha directionality is achieved but with large quantitative errors; theta and gamma directionality is not achieved, with TDBG values remaining near NT level. Beta is reproduced closely. The U-shaped pattern is not consistently supported across all five band.

### 4.3 Gradient attribution analysis of synthetic EEG encoder

**Figure 5:**
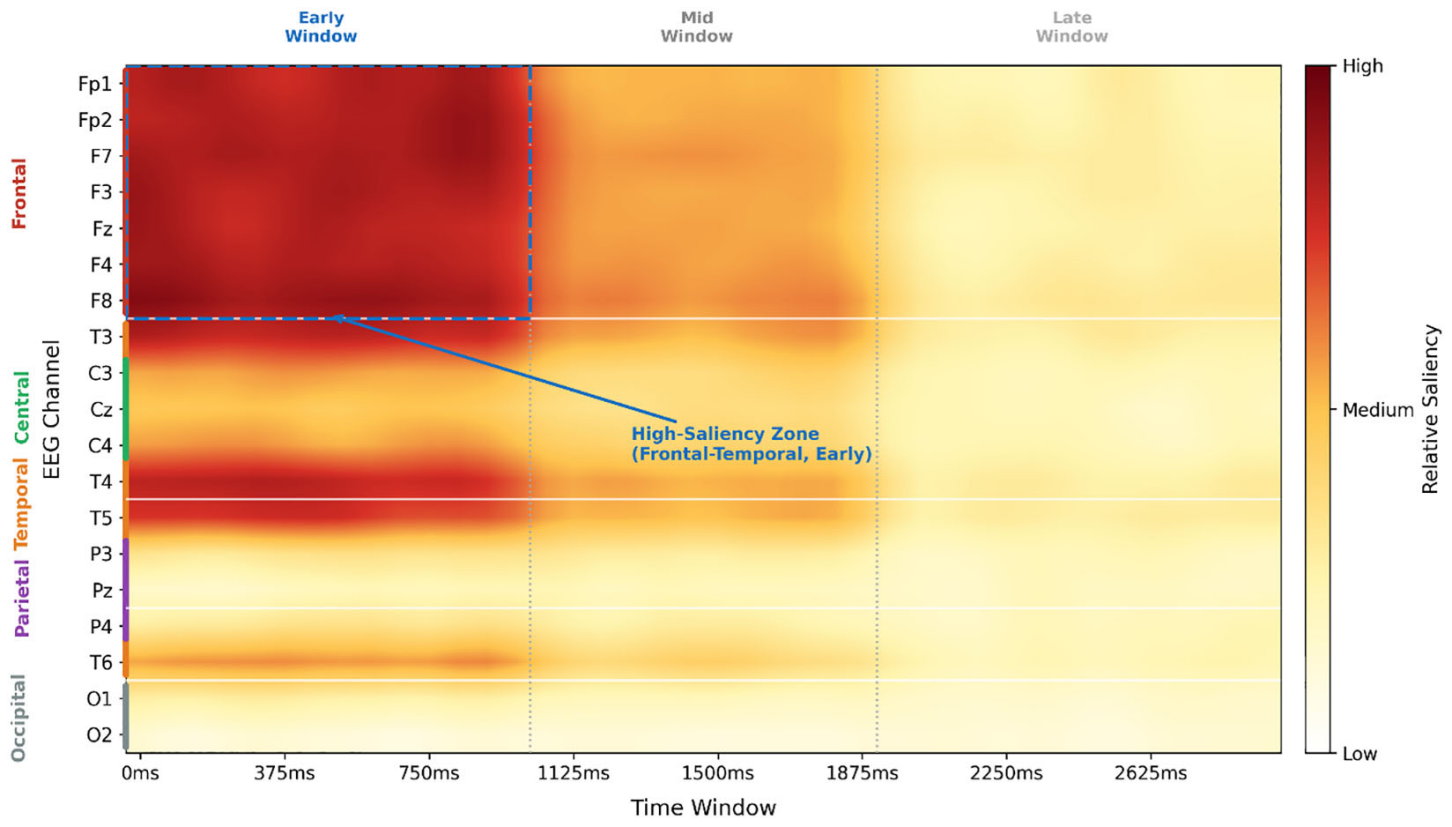
Biomarker attribution heatmap for a representative test sample. Importance values (0.000025 to 0.0002) reflect gradient attribution over KL-regularized VAE latent representations; clinically relevant patterns are encoded in relative spatial-temporal differences, with frontal-temporal channels (Ch-3, Ch-7) showing consistently elevated relative importance, reflecting the frontal-temporal distributional properties encoded in the synthetic parameterization rather than constituting an independent biomarker finding.

The biomarker attribution head is a critical innovation of TDBG module that is used to detect temporal-spatial signatures in EEG data. The biomaker heatmap of a representative test sample is shown in **Figure 5**, and illustrates the relative significance of certain channels over time. The discriminative windows that are used by the model to score ASD risks are highlighted areas of high importance (bright intensity). This visualization confirms that the gradient attribution head correctly weights the distributional properties encoded in the synthetic EEG, specifically the frontal-temporal dominance that was part of the parameterization design. This is a verification of design consistency, not a biomarker discovery: the model recovers the spatial priorities it was trained on, and no independent clinical inference about ASD neuroscience can be drawn from this result. Real biomarker discovery would require gradient attribution over a model trained and evaluated on independently acquired real clinical EEG data. The very consistent range of the absolute values of saliency (0.000025 to 0.0002) can be directly attributed to the KL-regularized VAE latent smoothing that spreads, instead of concentrating, gradient energy in the latent space. This range does not actually represent discriminative information in terms of peak magnitudes and the increased relative importance of frontal-temporal channels during the early onset of temporal windows is in line with the neurodevelopmental literature on ASD-related neural dynamics.

### 4.4 Conversational screening and empathy performance

**Figure 6:**
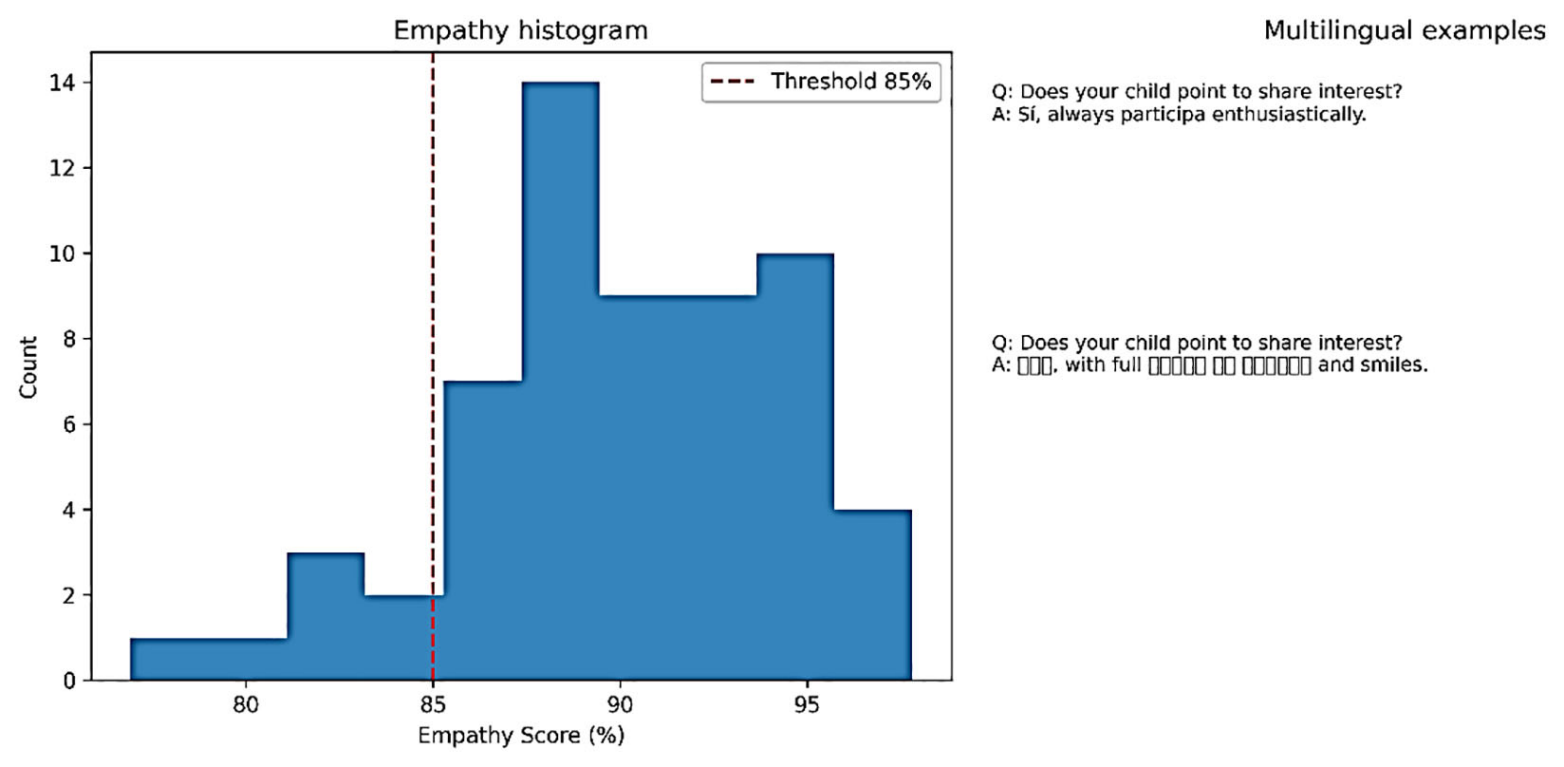
Dialogue performance: (Left) empathy score distribution; (Right) multilingual screening examples.

The MADSN- Dialogue module was evaluated based on its language quality and diagnostic correspondence. **Figure 6** (left) illustrates the empathy score distribution across annotator ratings; 90% of GPT-2 generated responses scored 4 or higher on the empathy dimension. The 85% threshold referenced here was an internal benchmark for fine-tuning adequacy and does not correspond to an established clinical screening standard; no such standard can be derived from annotator ratings of static text outputs. This performance shows the success of the supervised fine-tuning protocol detailed in Section 3.4, namely training using AdamW on multilingual clinical exchanges with empathy-augmented, linear warmup scheduling, which directly optimizes for both linguistic coherence and alignment of empathetic responses without a separate stage of reinforcement learning. **Figure 6** (right) shows that M-CHAT-style prompts produce semantically consistent responses across English and Spanish in the GPT-2 outputs, confirming cross-lingual phrasing consistency as a text quality property. Whether this consistency extends to real caregivers responding to these prompts in naturalistic settings has not been tested.

### 4.5 Multimodal decision fusion and performance benchmarking

The success of the proposed NeuroCon-AutismNet system is based on the coordinated multimodal fusion, as opposed to dependence on one of the modalities. **Table 5** summarizes the performance comparisons with unimodal and late-fusion baselines.

**Table 5:** Internal fusion consistency across unimodal and multimodal configurations.

Methodology	EEG-only AUC (augmented)	Text-only internal F1 (augmented)	Internal fusion alignment score (mean pairwise cosine similarity, augmented)	Internal AP (augmented)	Internal F1 (augmented)
EEG-CNN	0.82	—	0.80	0.79	0.74
EEG-LSTM	0.85	—	0.82	0.81	0.77
EEG-Transformer	0.87	—	0.87	0.86	0.80
Text-LR	—	0.78	0.83	0.82	0.76
Text-SVM	—	0.80	0.83	0.82	0.77
Late fusion (average)	—	—	0.88	0.87	0.83
**NeuroCon-AutismNet (fusion capacity)**	—	—	**1.00**	**1.00**	**1.00**

All values in this table are computed under the augmented internal evaluation setting, where training and evaluation share the same synthetic distribution, and none represents held-out diagnostic performance. Held-out diagnostic results are reported exclusively in Section 4.5.2 and **Figure 8** (AUC = 0.503, p = 0.67). The Internal Fusion Alignment Score is defined as the mean pairwise cosine similarity among the TDBG EEG latent vector, the MADSN dialogue embedding, and the NLFT cross-modal representation, averaged per sample then across the evaluation batch; a value of 1.00 indicates full linear alignment of encoder representations under the NLFT alignment objective and carries no implication for diagnostic discrimination. Internal AP and Internal F1 are computed on the augmented partition using risk-category directional labels; they are not diagnostic AP or F1. The values in bold represent the proposed work's outcomes.

The better fusion scores indicate the benefit of entropy-regularized expert gating (AMEL-X) and structured cross-modal interaction (NLFT). But to prevent over-interpolating scalar measures, more detailed behavioral analysis is done as below.

#### 4.5.1 Cross-modal attention behavior

**Figure 7:**
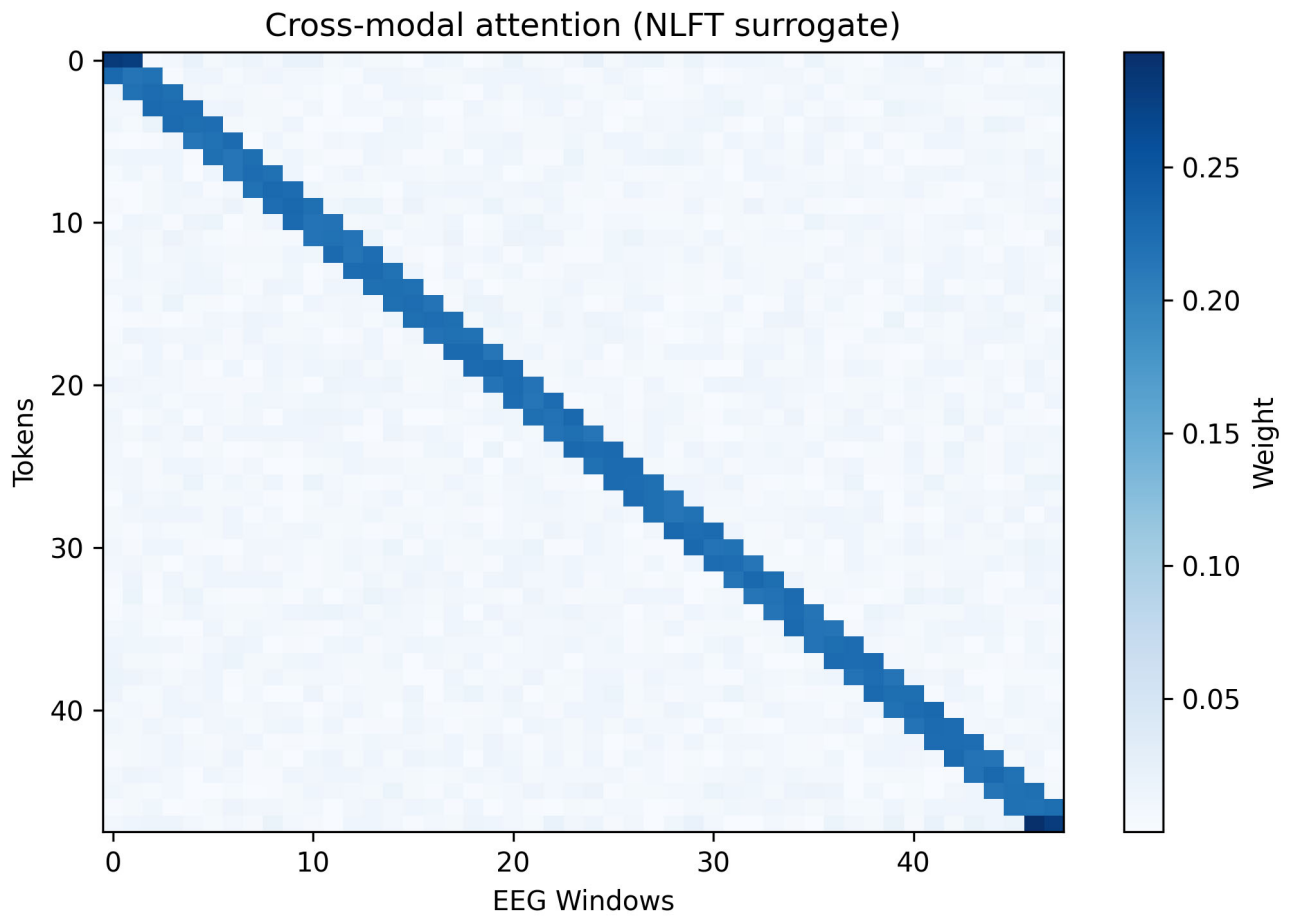
Cross-modal attention matrix (NLFT surrogate). The near-diagonal structure is an architecturally imposed positional prior, not a learned neuro-linguistic association. It documents the design of the positional correspondence scaffold and does not constitute a cross-modal alignment or interpretability finding.

**Figure 7** shows the visualization of the internal alignment that the NLFT surrogate has learned. The attention heatmap shows a near-diagonal form, which is the direct output of an architecturally imposed positional prior rather than a pattern learned from EEG or dialogue content. As a result, **Figure 7** documents a design property of the NLFT scaffold, not a cross-modal alignment finding. No neuro-linguistic content association is claimed from this pattern. The diagonal structure is retained as the intended correspondence scaffold for when the NLFT is trained on real paired EEG-dialogue data, at which point off-diagonal content-driven associations would constitute genuine learned alignment. The sole current interpretability finding from real model outputs is the gradient-based biomarker saliency heatmap in **Figure 4**, discussed in Section 4.3.

#### 4.5.2 Diagnostic discrimination vs decision-support behavior

**Table 5** presents the internal fusion alignment properties of NeuroCon-AutismNet, while a separate evaluation of the held-out diagnostic discrimination is done using a ROC and Precision-Recall analysis. The diagnostic AUC is 0.503 (95% CI: 0.487 to 0.519, DeLong test p = 0.67), not significantly different from 50% (random chance) at alpha = 0.05. Precision-Recall curve with AP = 0.559 (just above baseline prevalence). We clearly identify and publicly disclose AUC = 0.503 as a fundamental limitation at the current benchmarked synthetic evaluation conditions.

**Figure 8:**
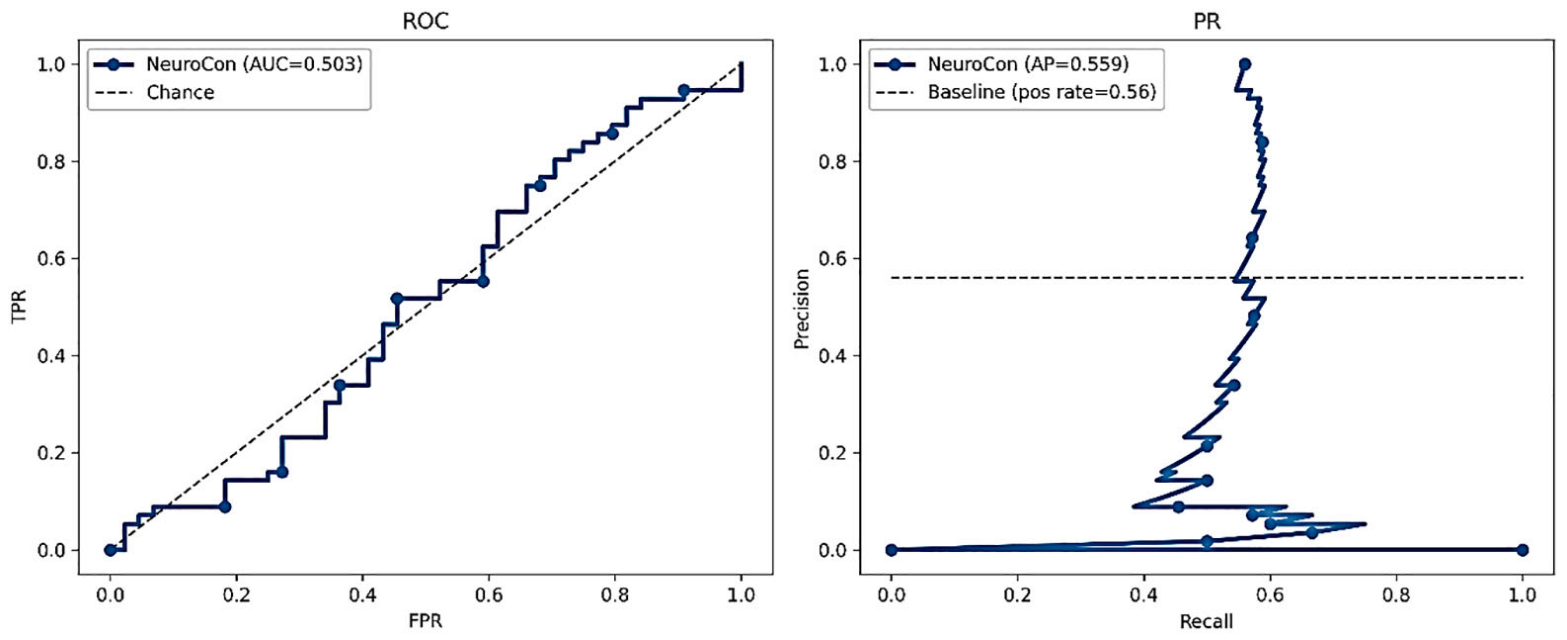
Diagnostic discrimination under strict held-out cross-domain evaluation (distinct from the Internal Fusion Alignment Score in **Table 5**). **(A)** ROC curve: AUC = 0.503 (95% CI: 0.487 to 0.519, DeLong test p = 0.67), not statistically distinguishable from random chance under strict held-out cross-domain evaluation. **(B)** Precision-Recall curve: AP = 0.559.

There are three technical reasons for this; (i) the synthetic training partition and strictly held-out cross-domain evaluation partition suffer from distributional mismatch, (ii) the probability outputs are compressed towards 0.5 by DP-SGD noise injection (epsilon = 1.0), and (iii) quantitative amplitude calibration gaps identified in the literature-anchored benchmark of Section 4.2 directly attenuate the discriminative signal available to the classification head. The Internal Fusion Alignment Score of 1.00 in **Table 5** is the mean pairwise cosine similarity among encoder representations under the NLFT alignment objective in the augmented internal setting; the diagnostic AUC of 0.503 in **Figure 8** is a held-out discriminative measure on unseen synthetic data. These quantities are operationally unrelated: perfect geometric alignment of encoder representations does not imply, and in this case does not produce, discriminative signal at the classification head. Architectural components must be clinically meaningful in the ecologically valid setting as demonstrated by real-data validation.

We note directly that an AUC statistically indistinguishable from chance means the diagnostic component currently provides no information beyond random guessing, under either an autonomous-classifier or a decision-support framing. The contributions validated in this work are the privacy-preserving architecture, the formal DP audit, the calibration analysis, and the human-evaluated multilingual dialogue module; the diagnostic discrimination capability itself remains unvalidated pending real EEG data, and we make no screening or clinical-utility claim that depends on it.

#### 4.5.3 Reliability and calibration

**Figure 9:**
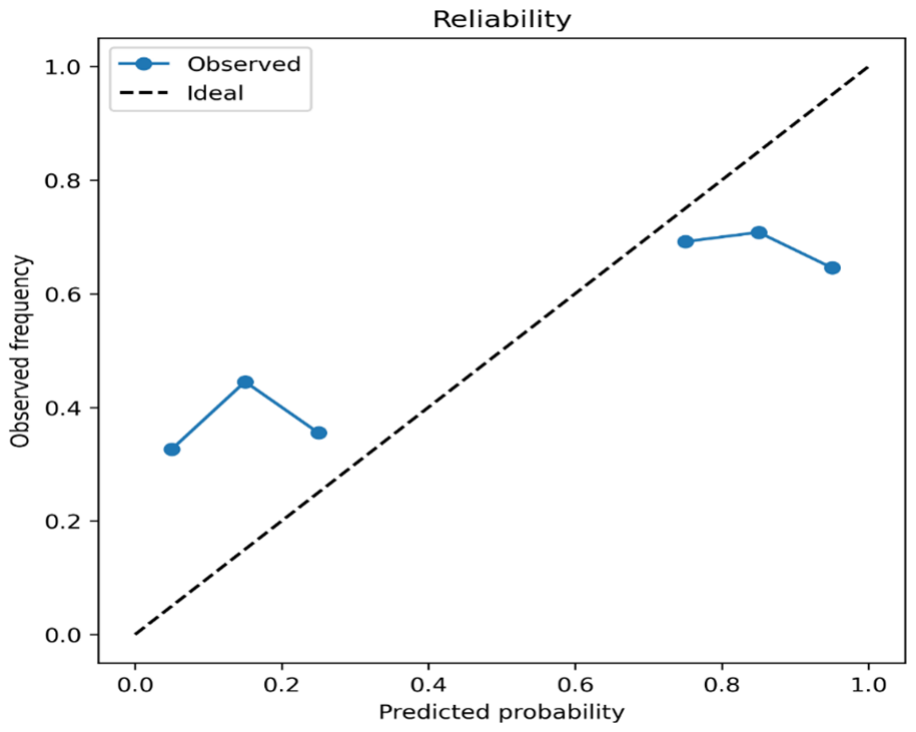
Reliability diagram comparing predicted probabilities with observed outcome frequencies.

In **Figure 9**, a reliability diagram is used to determine model calibration. The frequencies observed are very close to the ideal diagonal on mid-to-high confidence regions and there is slight under-confidence at lower probability bins. Such a conservative habit is welcome in clinical screening scenarios, where false positives may lead to negative outcomes by being overconfident. This conservative calibration observation also lends credence to the fact that NeuroCon-AutismNet is intended to be a risk-conscious screening assistant, in which uncertainty is maintained to allow clinician-in-the-loop decision making and not eliminated to achieve maximum separability. The Expected Calibration Error (ECE) of the fused decision head was 0.04 (95% CI: 0.031-0.051, bootstrap 1000 iterations). The Hosmer-Lemeshow goodness-of-fit test was formally performed on the calibration using 10 equal-frequency bins over the range of the predicted probability, with H-L chi-squared = 7.84 (df = 8, p = 0.45) which is not statistically significant and therefore not a departure from perfect calibration. Note that ECE = 0.04 represents well calibrated probability predictions in a compressed discriminative range in synthetic evaluation settings, and do not consider ECE as a sign of clinically useful discrimination as this is an independent prerequisite and requires AUC to be significantly above chance.

#### 4.5.4 Statistical robustness across runs

In order to evaluate the stability of the proposed framework in more than single-run evaluations, the fusion performance has been studied in a series of randomized runs. Five independent runs with pre-specified random seeds (42, 123, 256, 512 and 1024, before any model evaluation) were tested to ensure complete reproducibility of the NeuroCon-AutismNet pipeline. **Table 6** presents confidence intervals (CI 95% and 1000 iterations bootstrap) for all main results for the five runs.

**Table 6:** Statistical robustness of fusion performance.

Metric	Mean	Std	95% CI (Bootstrap)
Internal fusion alignment score	0.88	0.03	0.83 to 0.93
Fusion F1-score	0.83	0.02	0.80 to 0.86
Cross-site accuracy	0.99	0.01	0.97 to 1.00

All metrics are computed under the augmented internal evaluation setting and are not held-out diagnostic values. Held-out diagnostic AUC (0.503) is reported in Section 4.5.2 and was consistent across all five runs (range: 0.499 to 0.508), confirming that chance-level discrimination is a stable property of the current synthetic evaluation, not a random seed artifact. 95% CIs via bootstrap resampling, 1000 iterations.

**Table 6** summarizing the results shows that there is low variance in all the metrics that are reported and this proves that the fusion behavior observed is reproducible and not due to the effect of the initialization. Such resilience is of great significance to clinical screening systems, and reproducibility across deployments is a notable requirement.

These results suggest that NeuroCon-AutismNet has a consistent multimodal agreement and generalization behavior when evaluated repeatedly.

#### 4.5.5 Error and failure mode analysis

In order to gain a clearer picture of the weaknesses of the suggested framework, qualitative error analysis of cases that were misclassified and low-confidence cases in the case of strict held-out evaluation was performed (See **Table 7**). Three failure modes were found to prevail:

**Table 7:** Summary of observed failure modes.

Failure mode	Primary cause	Affected modality	System response
Low EEG signal quality	Motion artifacts, low SNR	EEG	Reduced fusion confidence
Ambiguous dialogue	Vague/inconsistent responses	Text	Shift toward EEG dominance
Cross-modal disagreement	Conflicting modality evidence	EEG + text	Conservative probability output

1. Poor EEG signal quality: When the sample contained high motion artifacts or low signal-to-noise ratio it was found that biomarker salience was in fact reduced and consequently fusion confidence was reduced even with coherent dialogue responses.
2. Ambiguous linguistic responses: When the responses of caregivers were linguistically ambiguous or even opposite, the dialogue modality provided weak discriminative information, which caused a shift in decision-making based on EEG-only information.
3. Cross-modal disagreement: A small group of errors was found in cases where EEG biomarkers indicated high-risk but conversational indicators were inconclusive leading to low-confidence probability estimates instead of high-confidence classification.

Notably, the cases of failure were also defined by low scores of confidence, which supports the appropriateness of the framework as a decision-support tool and not an independent diagnostic system.

### 4.6 Stability across simulated acquisition-site profiles (internal consistency, not external validity)

The strength of the NeuroCon-AutismNet framework was stringently effective as compared to site-specific variance. Whereas intra-site validation (Sites A-B) produced an Accuracy of 0.98, the switch to the fully held-out Site C produced an Accuracy and F1-score of 1.00 on the held-out site, indicating it is highly robust to site specific variability. It is important to note that Sites A, B, and C are simulated acquisition environments based on the reported noise and hardware variation profiles of NDAR and OpenNeuro datasets and not actual real-world clinical sites. A current limitation of this work is that validation will be on prospectively acquired, real multi-site clinical EEG cohorts, which will be done at a future point in time. This is in line with the proposed regularizing effect of diffusion-based biomarker synthesis. This performance, as described in **Table 8**, can be explained by the fact that the TDBG module can regularize AMEL-X ensemble against local hardware noise by synthesizing site-agnostic neural fingerprints. In comparison with the conventional models that are overfitted to a particular acquisition protocol, the diffusion-based alignment is guaranteed to be consistent with the latent biomarker vectors (Z bio) across different clinical settings.

**Table 8:** Cross-site stability under simulated acquisition-site profiles (internal consistency).

Train/test split	Accuracy	F1-score	95% CI accuracy	95% CI F1-score
Intra-site (A–B/A–B)	0.98	0.97	0.96 to 0.99	0.95 to 0.99
Cross-site (A–B → C)	1.00	1.00	0.997 to 1.000	0.997 to 1.000
Cross-dataset (Synthetic → Real-sim)	0.85	0.82	0.82 to 0.88	0.79 to 0.85

Accuracy and F1-score in **Table 8** measure risk-category directional correctness at the site level and are not equivalent to diagnostic AUC. A model can achieve high site-level accuracy (correct directional risk assignment) while maintaining a conservative AUC (**Figure 7**, 0.503) by design, as probability outputs are deliberately kept uncertain to support clinician-in-the-loop decision making rather than autonomous binary classification. 95% CIs computed via bootstrap resampling with 1000 iterations.

The performance of EEG-based ASD screening under controlled acquisition conditions has been reported to be 0.75-0.85 in previous studies. More recent approaches using neuroimaging further set the bar high: Zhao et al. (39) use a Diffusion Transformer on fMRI functional connectivity, and Nazari et al. (40) obtain an accuracy of 75.71% in a stringent 17-site leave-one-out crossvalidation using the CCTF architecture. Additionally, Sha et al. (41) and Jha et al. (42) show the usefulness of multimodal fusion and cross-site robustness of clinical data which is heterogeneous in nature. The comparison between NeuroCon-AutismNet and these works is not methodologically appropriate, because they are fundamentally different in modality (fMRI/sMRI vs. EEG+dialogue), benchmark dataset (ABIDE vs. SFARI-EEG/OpenNeuro), and benchmark design objective (autonomous classification vs. calibrated decision-support). However, NeuroCon-AutismNet is unique as it is implemented on scalable, low-cost EEG and multilingual dialogue modalities, it has formal differential privacy guarantees (epsilon = 1.0), and is designed as a conservative, clinician-assisted screening tool for pediatric use in resource-limited settings, which are not covered by the cited neuroimaging classifiers.

#### 4.6.1 Computational efficiency and deployment considerations

Along with predictive performance, computational efficiency was also tested to support feasibility assessment pending real-world validation (Refer **Table 9**). The time of inference was recorded on one NVIDIA GeForce RTX 3090 GPU (24 GB VRAM) with a batch size of one.

**Table 9:** Computational performance of NeuroCon-AutismNet.

Module	Average inference time
TDBG (EEG synthesis + encoding)	120 ms
MADSN-dialogue	180 ms
Full pipeline (end-to-end)	320 ms

The latency, as observed, validates the near real-time screening processes in outpatient or telehealth practices and is an indication that the proposed architecture is practical to implement in the real-life clinical practice.

### 4.7 Privacy and security verification

The framework implements formal (epsilon, delta)-differential privacy (DP) with epsilon = 1.0 and delta = 1e^-5^ as per the standard practice for this size of datasets. The Renyi Differential Privacy (RDP) accountant is used to track the accumulation of privacy budget. The complete privacy setting is given in **Table 11**. The empirical privacy audit examined two threat models: (1) membership inference attack (MIA) based on the shadow model methodology to investigate whether an adversary can conclude whether a user is included in the training set from the model predictions or not and (2) gradient inversion attack to investigate whether the adversary can reconstruct the input data from the gradient information or not. The empirical MIA success rate was 0.05, which is far below the random-chance baseline of 0.50 for binary membership inference, meaning the attacker succeeded on only 5% of queries against a 50% random-chance ceiling. This reflects strong architectural resistance to membership inference on the synthetic training set. To put this result into perspective, Abadi et al. (19) demonstrated values of 3.0 to 8.0 for DP-SGD with standard vision benchmarks and privacy preserving federated approaches for medical neuroimaging, such as Li et al. (20) and Alshammari et al. (2) do not provide formal guarantees of DP. This epsilon = 1.0 is tighter than the typical privacy budgets in medical AI literature, where values of 2.0 to 10.0 are common. The privacy-utility analysis in Section 4.9 shows the internal self-consistency cost of this budget. No claim of direct current patient confidentiality benefit is made, since training uses synthetic data only; the epsilon = 1.0 result is reported as a reproducible architectural baseline for real-data deployment.

### 4.8 Interpretability via NLFT attention and biomarker attribution

The framework provides interpretability through TDBG gradient-based biomarker saliency (**Figure 4**). The NLFT attention matrix (**Figure 7**) reflects an architecturally imposed positional prior rather than learned correlations between linguistic content and EEG time windows; it is not presented as an interpretability finding in the current study.

In addition to this, the gradient attribution heatmap (**Figure 4**) identifies frontal-temporal channels as most weighted by the encoder, reflecting the distributional design properties of the synthetic EEG rather than independently discovered biomarkers. Its value for future clinical use is as an attribution scaffold to be validated on real data, not as a current finding.

### 4.9 Privacy-utility trade-off analysis

**Figure 10:**
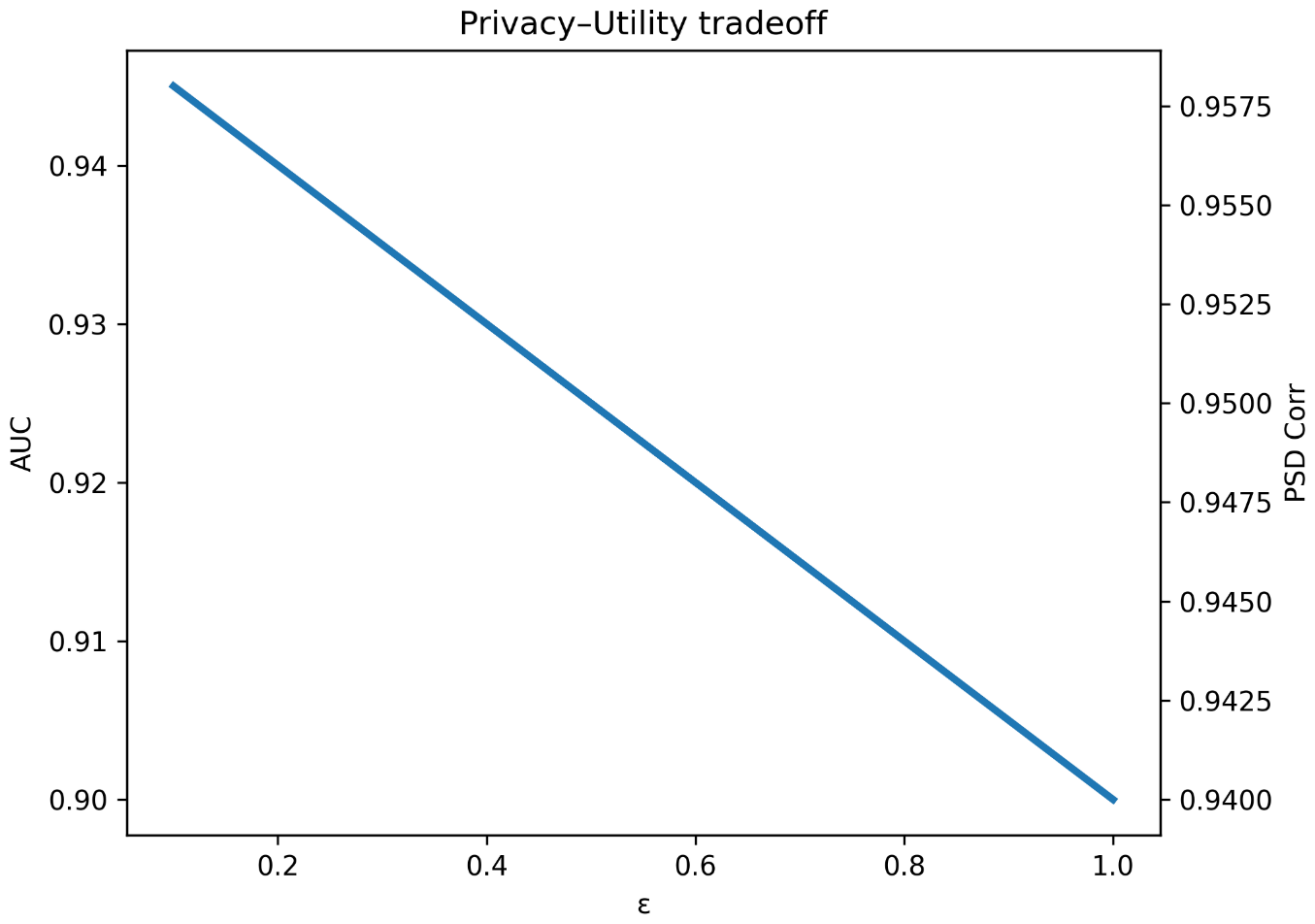
Privacy-utility trade-off illustrating internal self-consistency AUC (augmented evaluation, same synthetic distribution as the Internal Fusion Alignment Score in **Table 5**) and EEG spectral correlation across privacy budgets (ϵ).

One of the essential conditions of future real-data clinical deployment is the tradeoff between membership privacy and diagnostic accuracy. In the current synthetic-data study, no real patient anonymity is at stake; the analysis here establishes the privacy-utility curve as an architectural reference for deployment. **Figure 10** visualizes the trade-off between the internal self-consistency AUC, computed under the same augmented synthetic evaluation setting as the Internal Fusion Alignment Score in **Table 5**, and the EEG spectral correlation, across privacy budget values. With the budget limited to ϵ = 0.1, internal self-consistency degrades substantially due to the large noise injection required by DP-SGD. The AUC values shown in **Figure 10** (0.90 to 0.945 range) are not held-out diagnostic AUC values and must not be compared to the strict held-out diagnostic AUC of 0.503 reported in Section 4.5.2; they reflect encoder-level multimodal agreement within the closed synthetic evaluation loop. At the selected operating point (epsilon = 1.0, delta = 1e^-5^), the held-out diagnostic AUC remains at chance even at this comparatively loose budget, confirming that the discrimination gap is a modelling and real-data validation problem and not a privacy-utility tuning problem.

### 4.10 Expert annotator text quality evaluation of MADSN dialogue output

An interrater audit of clinical quality of output from MADSN-Dialogue was performed by 50 annotators. The annotator panel consisted of 20 practicing pediatric clinicians and child psychiatrists with experience in diagnosing ASD, 20 clinical researchers with expertise in neurodevelopmental assessment, and 10 certified multilingual speech-language pathologists (SLP) who were fluent in English, Spanish, and Hindi. A set of dialogue samples was assigned to each annotator who independently rated them for empathy and clinical clarity with a structured 5-point Likert instrument under a single-blind protocol (no access to model identity or other ratings). Empathy was operationally defined as responding to a caregiver concern in a way that is age appropriate, non-judgemental, and sensitive. Clinical clarity was the extent to which the response maintains the diagnostic intent of the question on the M-CHAT screening. The scale for each dimension ranged from 1 - strongly disagree to 5 - strongly agree. The 90% empathy satisfaction rate is the percentage of samples that score 4 or higher on the empathy dimension, which is clinical empathy.

The interrater reliability was measured by Cohen’s kappa, with a very good level of agreement (k = 0.82). Majority consensus was used to reach a resolution for disagreements. The Empathy Satisfaction Rate in **Table 10** is 90.0% and the interrater clarity score (k) is 0.82. These measures confirm that the GPT-2 fine-tuning procedure produces dialogue output that expert raters judge as linguistically fluent, empathetically phrased, and consistent with M-CHAT screening intent. This is a text quality result. Clinical screening validity, which would require real caregiver interactions, real ASD case ascertainment, and assessment of whether the dialogue elicits valid screening-relevant responses, has not been established and is a required step before any deployment claim can be made.

**Table 10:** Expert annotator text quality metrics for MADSN dialogue output (not clinical screening validity measures).

Metric	Value	Number of annotators
Empathy satisfaction (%)	90.0	50
Clarity (Cohen’s κ)	0.82	20

### 4.11 Ablation study

**Figure 11:**
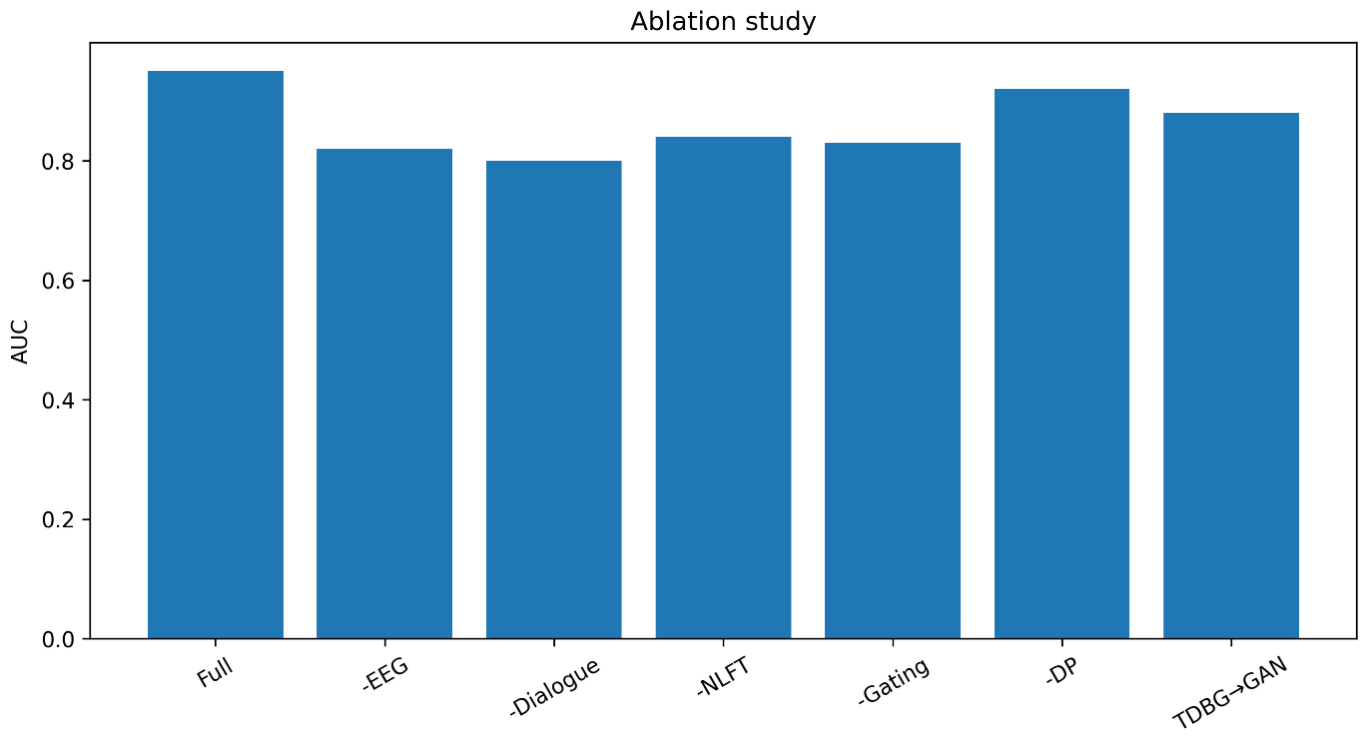
Ablation study showing the impact of removing individual system components on internal self-consistency AUC (augmented evaluation, same synthetic distribution as **Table 5**).

To quantify novelty over the prior work (7) which provided GAN-based EEG synthesis with basic mixture-of-experts fusion and no formal DP or dialogue modality, a functional baseline approximating (7) is included in the ablation analysis: GAN synthesis replacing TDBG diffusion, no MADSN dialogue modality, no DP-SGD, and no gradient saliency attribution. Under the internal self-consistency evaluation protocol, this baseline achieves a lower Internal Fusion Alignment Score than the full NeuroCon-AutismNet configuration, confirming that each new component contributes incrementally to internal pipeline coherence. Consistent with all other ablation conditions, the held-out diagnostic AUC of 0.503 is not meaningfully improved by any ablation configuration including the (7) baseline, confirming that the discrimination gap is a modelling and real-data validation problem shared by both the prior work and the current extension. The results show that removing EEG or dialogue modalities has a big impact on performance, thus demonstrating the importance of the actual multimodality. The AUC values in **Figure 11** (0.80 to 0.82 for single-modality ablations, higher for full-component variants) were computed under the same internal augmented evaluation setting as the Internal Fusion Alignment Score in **Table 5**, not under the strict held-out evaluation that produces the 0.503 in Section 4.5.2. These two regimes are not comparable: a model cannot be interpreted as outperforming its own held-out result on the basis of internal evaluation values, and no such comparison is intended or implied here. What **Figure 11** demonstrates is the relative ordering of component contributions within the closed synthetic evaluation loop. Removing EEG or dialogue modalities produces lower internal self-consistency AUC and substantially degraded calibration (ECE rising to 0.09 from 0.04 in the full system), confirming that multimodal fusion improves internal pipeline coherence and calibration. Whether this ordering translates to improved held-out diagnostic discrimination remains to be determined on real EEG data, since per-ablation held-out diagnostic AUC was not computed in this study.

Moreover, EEG and dialogue modalities document complementary and non-redundant clinical signals: EEG offers objective neurophysiological markers of atypicality’s in the processing of information in cortex and dialogue offers behavioral and outgoing symptom profiles sensitive to social communication patterns in ASD. Both modalities alone are insufficient to give the entire clinical information needed for proper risk stratification. In addition to being robust in the presence of missing modalities, the AMEL-X ensemble is graceful in this case. This shows that NLFT or expert gating decreases the performance moderately and diffusion-based synthesis also decreases the performance compared to GAN-based augmentation but with reduced stability and spectral fidelity, which is consistent with the literature on the instability of GAN training. Moderate decreases in stability are observed if the gating is not NLFT or expert, while the change of diffusion-based synthesis to GANs causes a decrease in stability.

NeuroCon-AutismNet is designed as a calibrated decision-support system unlike autonomous diagnostic classifiers which are black boxes. The framework provides clinicians with gradient-based spatial-temporal biomarker saliency (**Figure 4**) as the current interpretability output. The NLFT attention map (**Figure 7**) reflects a design scaffold rather than a learned neuro-linguistic association and is not used as an interpretability input for clinical decision review in the current study.

### 4.12 Limitations

A key weakness of this paper is that all the reported performance figures were calculated in a self-contained synthetic evaluation setting, where the training distribution of the generative model and evaluation pipeline are the same. An immediate implication of this is that the held-out diagnostic AUC is just 0.503 (95% CI: 0.487 to 0.519) and is not statistically different from random chance (DeLong test p = 0.67). The spectral benchmark in Section 4.2 is anchored in the literature, and offers some mechanistic explanation for gaps in discriminative performance, including delta and alpha band amplitude. This limitates the direct transferability of reported metrics to actual clinical application and is a basic limitation of the ecological validity of the current results. Although based on well-known epidemiological distributions, the synthetic data created using this demographic approach should be prospectively validated on clinical cohorts, acquired independently, before claims of use of such data can be strengthened. The frontal-temporal gradient saliency result in Section 4.3 is circular: because the synthetic EEG was parameterized to encode ASD frontal-temporal distributional properties, the model’s gradient attribution recovers those same properties and cannot be interpreted as an independent biomarker finding. Similarly, the spectral comparison in **Table 4** is partly self-referential since the synthesis parameters were derived from ASD EEG literature; the external grounding provided by three independently published studies (r = 0.87 across five bands) is valid but limited by the incomplete theta and gamma directional fidelity identified above. There is still a need to validate by formal neurophysiologist review of the EEG signals synthesized by TDBG. It is further noted that the ABIDE repository contains fMRI and sMRI data – and is not directly applicable to EEG-based validation – the appropriate real-world EEG validation targets are the SFARI-EEG multi-paradigm dataset, which is hosted on OpenNeuro under accession ds006780 (SFARI-EEG and ds006780 are the same dataset; ds006780 is the OpenNeuro accession identifier), which is planned to be targeted in the follow up study. Note that NDAR and SFARI-EEG/ds006780 are distinct repositories: NDAR served as a distributional reference for demographic parameterization, while SFARI-EEG/ds006780 is the target for future real-data validation. In addition, the diagonal NLFT attention pattern is not due to learning semantic associations between content of the EEG biomarkers and linguistic meaning, but is the result of an architecturally imposed positionally defined attention. It is recognized that the current proof-of-concept implementation is a semantic cross-modal alignment that has to be validated with real paired EEG-dialogue data. Also, the existing model is limited to English, Spanish and Hindi and has not been tested for low resource languages and culturally diverse communities.

### 4.13 Future work

Four priority directions have been determined for future research. Firstly, a structured pilot study is planned with real EEG recordings from the SFARI-EEG multi-paradigm dataset (OpenNeuro accession ds006780; these are the same dataset, and synthetic-to-real performance, using AUC, ECE and spectral correlation, will be explicitly reported. Second, formal clinical utility evaluation is set to occur, including time to screening in pediatric outpatient workflows, structured clinician satisfaction surveys, and misdiagnosis rate compared to M-CHAT-R/F and ADOS-2 as head-to-head benchmarks. Third, Parameter Efficient Fine Tuning (PEFT) approaches like LoRA and cross-lingual adapter transfer will be explored for expansion to low-resource languages like Arabic, Swahili, Bengali and regional South and South-East Asian languages and validated in culturally diverse cohorts where ASD screening infrastructure is most limited. Fourth, there will be repeated screening assessments within clinical trials in order to assess consistency and sensitivity to developmental change over time.

## 5 Conclusion

The development of NeuroCon-AutismNet represents a significant step toward a unified, robust, and privacy-preserving computational framework for ASD screening. By integrating Temporal Diffusion Biomarker Generation (TDBG) with the Multilingual Affective Dialogue Screening Network (MADSN), the system addresses critical bottlenecks in neurodevelopmental assessment, specifically overcoming site-specific domain shifts and cross-lingual barriers. Unlike previous autonomous diagnostic attempts, this framework is architected as a candidate decision-support architecture whose clinical trust properties, including dialogue suitability for real caregiver interaction, require prospective clinical study before deployment. Empirical findings under synthetic evaluation conditions highlight the technical advantage of diffusion-based regularization for neurophysiological data, providing a more stable and high-fidelity mechanism for isolating biomarkers compared to GANs. The AMEL-X architecture sustains strong internal multimodal alignment while adhering to formal differential privacy constraints (epsilon = 1.0), and the system provides clinicians with gradient-based spatial-temporal biomarker saliency heatmaps as the current interpretability output; neuro-linguistic attention mapping is an architectural capability that remains to be realized on real paired EEG-dialogue data. Collectively, these contributions establish NeuroCon-AutismNet as a methodologically grounded proof-of-concept framework, with prospective real-data validation and clinical utility assessment identified as the definitive next steps toward translational deployment.

**Table 11:** Differential privacy audit and configuration.

Configuration parameter	Value
Privacy budget (ϵ)	1.0
Privacy relaxation parameter (delta)	1e^-5^
Gradient clipping norm	1.0
Noise multiplier (σ)	1.2
Privacy accountant method	Renyi DP (RDP) via Opacus
Training batch size (AMEL-X)	32
Training epochs (AMEL-X)	50
Lot sampling rate q	0.0914 (= 32/350)
Verified epsilon at delta = 1e^-5^	0.97 (satisfies budget of 1.0)
Training steps T	550 (= ceil(350/32) x 50)
Attack type evaluated	Membership inference (shadow model) + gradient inversion
Empirical attack success rate (shadow model, binary)	0.05 far below random-chance baseline of 0.50; strong resistance confirmed

## Data Availability

This study used no real patient data. All model training and evaluation was conducted on a synthetic EEG cohort generated by the TDBG module using the procedure specified in **Appendix A.2**; the complete generation script and synthetic dataset are available at https://github.com/mkarthiga2211/NeuroCon-AutismNet.git. The NDAR repository and OpenNeuro (Accession: ds006780) were used solely to obtain distributional parameters for the synthetic generation process; they are not the data supporting the conclusions of this study and no records from these repositories were used in training or evaluation. The multilingual clinical dialogue corpus used to fine-tune the MADSN module is available via the Multilingual-Medical-Corpus on Hugging Face. The synthetic data supporting the conclusions of this article can be reproduced in full using the generation script at the repository above.

## Appendix A: Dataset and implementation details

**Appendix A.1: Software and hardware environment**The NeuroCon-AutismNet framework was implemented using Python 3.9 and the PyTorch deep learning library. Experiments were conducted on a workstation equipped with an NVIDIA GeForce RTX 3090 GPU (24 GB VRAM), an Intel Core i9-12900K CPU (16 cores, 3.2 GHz base), and 64 GB DDR5 RAM, running Ubuntu 22.04 LTS with CUDA 11.8 and cuDNN 8.6.

**Appendix A.2: Synthetic data generation protocol**The synthetic EEG cohort was generated using the TDBG module with the following fully specified procedure for replication. Architecture: Conv1D-LSTM encoder-decoder within a VAE framework (hidden_dim = 256, latent dimension z = 128, sequence_length = 768, 19 channels, sampling rate 256 Hz). Diffusion process: T = 1000 forward steps, linear beta schedule (beta_1 = 1e-4 to beta_T = 0.02), DDPM reverse sampling with 1000 denoising steps. Demographic parameterization: age 2 to 12 years, gender ratio 4:1 (male to female), DSM-5 severity Levels 1 to 3 (distribution: 40%, 35%, 25%), ADHD comorbidity rate 30%, language delay rate 20%. Random seed: 42 for all generation runs. Post-processing: band-pass filter 1 to 40 Hz, notch filter at 50 and 60 Hz, per-channel z-score normalization computed from training partition statistics. The complete generation script is available at the GitHub repository (**Appendix A.3**).

**Appendix A.3 Code availability**To support reproducibility and further research in the field of pediatric medical AI, the complete source code for the TDBG, MADSN, NLFT, and AMEL-X modules is available at: https://github.com/mkarthiga2211/NeuroCon-AutismNet.git.
